# Regulation of Exopolysaccharide Production by ProE, a Cyclic-Di-GMP Phosphodiesterase in *Pseudomonas aeruginosa* PAO1

**DOI:** 10.3389/fmicb.2020.01226

**Published:** 2020-06-05

**Authors:** Qishun Feng, Stephen Dela Ahator, Tian Zhou, Zhiqing Liu, Qiqi Lin, Yang Liu, Jiahui Huang, Jianuan Zhou, Lian-Hui Zhang

**Affiliations:** ^1^Guangdong Province Key Laboratory of Microbial Signals and Disease Control, Integrative Microbiology Research Centre, South China Agricultural University, Guangzhou, China; ^2^Guangdong Laboratory for Lingnan Modern Agriculture, South China Agricultural University, Guangzhou, China

**Keywords:** *Pseudomonas aeruginosa*, c-di-GMP, phosphodiesterase, enzymatic properties, exopolysaccharide, Pel and Psl

## Abstract

The ubiquitous second messenger c-di-GMP is involved in regulation of multiple biological functions including the important extracellular matrix exopolysaccharides (EPS). But how c-di-GMP metabolic proteins influence EPS and their enzymatic properties are not fully understood. Here we showed that deletion of *proE*, which encodes a protein with GGDEF-EAL hybrid domains, significantly increased the transcriptional expression of the genes encoding EPS production in *Pseudomonas aeruginosa* PAO1 and changed the bacterial colony morphology. Our data showed that ProE is a very active phosphodiesterase (PDE), with a high enzyme activity in degradation of c-di-GMP. Interestingly, the optimal activity of ProE was found in the presence of Co^2+^, unlike other PDEs that commonly rely on Mg^2+^ or Mn^2+^ for best performance. Furthermore, we identified three widely conserved novel residues that are critical for the function of ProE through site-directed mutagenesis. Subsequent study showed that ProE, together with other three key PDEs, i.e., RbdA, BifA, and DipA regulate the EPS production in *P. aeruginosa* PAO1. Moreover, by using the GFP-fusion approach, we observed that these four EPS associated-PDEs showed a polar localization pattern in general. Taken together, our data unveil the molecular mechanisms of ProE in regulation of EPS production, and provide a new insight on its enzymatic properties in degradation of c-di-GMP.

## Introduction

The cyclic dinucleotide (c-di-GMP) is a conserved second messenger in many bacteria species (Romling et al., 2013). It plays important roles in regulation of biofilm formation, motility, virulence, development, and cell cycle progression (Jenal and Malone, 2006; Hengge, 2009; Jenal et al., 2017). The synthesis and degradation of cyclic di-GMP is controlled by two classes of enzymes, i.e., the GGDEF domain containing diguanylate cyclase (DGC), EAL or HD-GYP domain containing phosphodiesterase (PDE). C-di-GMP acts by binding to specific effectors, including PilZ containing protein (Xu et al., 2016), inactive GGDEF or EAL domains (Navarro et al., 2009; Whitney et al., 2012), riboswitches (Sudarsan et al., 2008) and transcription factors (Tao et al., 2010; Baraquet et al., 2012), to regulate downstream genes associated with different biological functions. Among the products regulated by c-di-GMP, EPS are the key component of extracellular matrix involved in surface adhesion, cell-cell interactions and biofilm formation (Bazaka et al., 2011; Koo et al., 2013; Matthysse, 2014), and are regulated by c-di-GMP binding proteins such as Alg44, PelD, and BcsA (Lee et al., 2007; Merighi et al., 2007; Morgan et al., 2014).

The opportunistic human pathogen *Pseudomonas aeruginosa* can cause severe infections in cystic fibrosis patients and immunocompromised individuals (Lee and Zhang, 2015; Ahator and Zhang, 2019). The pathogen produces at least three polysaccharides, including alginate, Psl, and Pel (Ryder et al., 2007). Among them, alginate is the predominant extracellular polysaccharide of the extracellular matrix in mucoid strains (Hentzer et al., 2001), while Pel and Psl polysaccharides were primarily utilized for biofilm formation by non-mucoid strains (Wozniak et al., 2003). *P. aeruginosa* PAO1 has two operon which can synthesis Pel and Psl, respectively (Friedman and Kolter, 2004). The production of Pel and Psl are regulated by transcription factors such as FleQ and AmrZ (Hickman and Harwood, 2008; Jones et al., 2014), and DGCs or PDEs (Liang, 2015).

*Pseudomonas aeruginosa* PAO1 encodes a total of forty-one proteins which are involved in the metabolism of c-di-GMP (Kulasakara et al., 2006). During the last decade, more than half of c-di-GMP metabolic proteins have been functionally characterized (Hickman et al., 2005; Hoffman et al., 2005; Kulasekara et al., 2005; Merritt et al., 2007; Roy et al., 2012; Basu Roy and Sauer, 2014). Previous studies showed that Pel was regulated by DGCs WspR, YfiN, SadC, and RoeA (Guvener and Harwood, 2007; Merritt et al., 2007, 2010; Malone et al., 2010, 2012), and by PDEs BifA and RbdA (Kuchma et al., 2007; An et al., 2010). Interestingly, Psl can act as a signal to activate SiaD and SadC, two DGCs, thus increasing the production of Psl and other components of the biofilm in *P. aeruginosa* PAO1 (Irie et al., 2012). A comprehensive study showed that more than half of these c-di-GMP metabolic proteins can influence the EPS production in PA14 (Ha et al., 2014).

However, the enzymatic properties and underlying mechanism with which c-di-GMP metabolic proteins modulate EPS production remain to be further studied. In this study, we identified the gene *PA5295*, designated as *proE* for its role as an important phosphodiesterase regulator of EPS. The *proE* gene encodes a dual-domain protein consisting of GGDEF-EAL domains. Genetic and biochemical analyses revealed the role of *proE* as a highly active PDE, which negatively regulates EPS production in strain PAO1. Three novel conserved residues of ProE were identified to play key roles in c-di-GMP metabolism. Our *in vitro* analysis shown that purified ProE was more active in degradation of c-di-GMP than the previously characterized highly active PDE RocR (Kulasakara et al., 2006; Rao et al., 2008; Chen et al., 2012). Furthermore, we provided evidence that ProE with other three PDEs together controlled the EPS production in *P. aeruginosa*. Our subcellular localization analysis indicated that these EPS-associated proteins were more or less localized in cell poles.

## Materials and Methods

### Bacterial Strains, Plasmids, Media and Growth Conditions

Bacterial strains and plasmids used in this study are listed in Supplementary Table S1. Bacteria were routinely maintained at 37°C in Lysogenic Broth (LB). Antibiotics at the following concentrations were added when necessary: gentamicin, 50 μg/ml for *P. aeruginosa*; and gentamicin, 25 μg/ml; kanamycin, 50 μg/ml for *Escherichia coli*.

### Construction of In-Frame Deletion Mutants and Complementation

The plasmids and primers used in this study are listed in Supplementary Tables S1, S2, in the Supplementary Material, respectively. To generate the *proE* deletion mutant of *P. aeruginosa*, two PCR fragments flanking *proE* were amplified. After purification with NucleoSpin Gel and PCR Clean-up kit (Macherey Nagel), the two flanking fragments were ligated with the linear vector pK18 (digested with *Eco*RI and *Bam*HI) by One Step Cloning Kit (Vazyme Biotech, Nanjing, China). The resultant construct was transformed into *E. coli* DH5α competent cells by heat shock at 42°C and introduced into strain PAO1 through triparental mating. In frame-deletion was performed as described previously (An et al., 2010). The generated *proE* deletion mutant was confirmed by PCR and DNA sequencing. Single-deletion mutants Δ*proE*, Δ*fleQ*, Δ*rbdA*, Δ*bifA*, Δ*dipA*, Δ*pelA*, Δ*pslA*, and Δ*PA5294*, double-deletion mutants Δ*proE*Δ*pelA*, Δ*proE*Δ*pslA*, Δ*fleQ*Δ*pel*A, Δ*fleQ*Δ*pslA*, and Δ*fleQ*Δ*proE*, and the triple-deletion mutants Δ*proE*Δ*pelA*Δ*pslA*, Δ*fleQ*Δ*pel*AΔ*pslA* were generated by the same procedure using corresponding primers (Supplementary Table S2).

For *in trans* complementation, the coding region with native promoter was amplified by the primers listed in Supplementary Table S2, the PCR products were cloned into the plasmid pBBR1-MCS5 digested by *Eco*RI and *Bam*HI. The resultant construct was mobilized into *E. coli* DH5α and sequenced before introducing it into the corresponding mutants by tri-parental mating and then confirmed by PCR analysis.

### Colony Morphology Assay

Overnight cultures were diluted 1/1000 in ddH_2_O. One microliter of cells were spotted onto T-agar plates (10 g/l tryptone, 1% agar) supplemented with 40 mg/ml Congo red and 15 mg/ml Coomassie brilliant blue R-250 (Sigma-Aldrich, United States) and incubated at 25°C for 2 days prior to observation and taking photographs.

### RNA Extraction and Quantitative Real-Time PCR (qRT-PCR)

Bacteria were grown in LB medium and harvested at the mid-exponential phase (OD_600_ about 0.5), and RNA samples were prepared using the RNAprep Pure Cell/Bacteria Kit (TIANGEN, Beijing, China), following the manufacturer’s protocol. The integrity and purity of RNA was determined by agarose gel electrophoresis and the concentration was measured by NanoDrop 2000C (Thermo Fisher Scientific, Waltham, MA, United States). The first-strand cDNA was reversely transcribed by using the FastKing RT kit (with gDNase) (Tiangen Biotech, Co., Ltd., Beijing, China) with 1.5 μg RNA. Quantitative real-time PCR (qRT-PCR) was performed on a QuantStudio 6 Flex Real-Time PCR System using PowerUp SYBR green master mix (Applied Biosystems, United States) with the following PCR procedure: 50°C for 2 min, 95°C for 2 min, (95°C, 15 s; 60°C, 1 min) × 40 cycles. The experiment was repeated three times, each time with triplicates. All the primers are listed in Supplementary Table S2. The relative expression levels of the target genes were normalized to the housekeeping gene *rplU* and the gene expression level was calculated by using 2^–ΔΔ*CT*^ method (Kuchma et al., 2007).

### Protein Cloning, Expression, and Purification

The DNA fragments encoding *proE*, *rocR*, and *wspR* were amplified with the primers listed in Supplementary Table S2 and cloned into the expression vector pET28b (+) (Novagen) between the *Bam*HI and *Hin*dIII restriction sites. For protein expression, 10 ml overnight culture of the expression strains of ProE, RocR, and WspR were added to 1 L of LB medium, respectively. The bacterial culture was grown at 37°C until it reached an OD_600_ about 0.5 before addition of 0.5 mM isopropyl-β-D-thiogalactopyranoside (IPTG) at 18°C overnight. The cell pellet was resuspended in 25 ml lysis buffer, which consists of 50 mM Tris–HCl (pH 8.0), 200 mM NaCl. The cells were then lysed by sonication, after centrifugation at 12,000 rpm for 1 h, the supernatant was filtered by 0.45 μm filter membranes (Pall Corporation, United States) and then incubated with 5 ml of Ni^2+^-nitrilotriacetic acid resin (Clontech, Japan) for 2 h on ice. The resin was washed with 50 ml of washing buffer (lysis buffer with 20 mM imidazole). The proteins were eluted using a stepped gradient method with the elution buffer containing 50 mM Tris–HCl (pH 8.0), 200 mM NaCl, and 50 mM, 100 mM, 200 mM, 300 mM, or 400 mM imidazole. After sodium dodecyl sulfate-polyacrylamide gel electrophoresis analysis, fractions with purity higher than 95% were pooled together and desalted using a HiPrep 26/10 Desalting column (GE Healthcare, United States). Proteins were concentrated using an Amicon concentrator (Merck Millipore, Germany) and flash-frozen in liquid nitrogen, then the protein samples were stored at -80°C prior to use.

### Enzymatic Activity Assay

Enzyme activity analyses were performed following the methods described previously, with minor modifications (An et al., 2010). For PDE activity assay, 0.08 μM ProE or RocR were added to reaction buffer containing 50 μM c-di-GMP, 100 mM Tris–HCl (pH 8.0), 20 mM KCl, 5 mM MgCl_2_, in a final volume of 50 μl. The reaction mixture was incubated at 37°C for 20 min, then stopped by adding 1/10 volume of 1 M CaCl_2_ and heating at 95°C for 10 min. The PDE activity was assessed by monitoring the formation of the product 5′-pGpG from the hydrolysis of c-di-GMP. To test the effect of GTP, a final concentration of 50 μM GTP was added into the reaction mixture and incubated for 10 min at room temperature prior to reaction, then the enzyme reaction was performed as described above.

For DGC activity assay, 500 μM GTP and 5 μM ProE or WspR were added to reaction buffer containing 75 mM Tris–HCl (pH 7.8), 250 mM NaCl, 25 mM KCl, 10 mM MgCl_2_ in a final volume of 50 μl. The reaction mixture was incubated at 37°C for 120 min. High-performance liquid chromatography (HPLC) analyses were performed by running the samples through a reverse-phase C18 column (YMC-Pack ODS-A, 250 × 4.6 mm, 5 μm) on a HPLC chromatographic system (Agilent 1260 Infinity, United States) at an injection volume of 10 μl, using the solvents and elution gradient as previous described (Chua et al., 2015), at a flow rate of 1.0 ml/min and with a detection wavelength of 254 nm.

### Site-Directed Mutagenesis

The constructs pBBR1-MCS5-*proE* and pET28b-*proE* were used as templates for site-directed mutagenesis. PCR was carried out by using Phanta Max Super-Fidelity DNA Polymerase (Vazyme Biotech, Nanjing, China) and relevant mutagenic PCR primers (Supplementary Table S2). The PCR products were treated with *Dpn*I to digest the methylated and hemimethylated DNA, then ligated by One Step Cloning Kit (Vazyme Biotech, Nanjing, China) prior to transformation of *E. coli* strain DH5α. The resultant mutations were confirmed by PCR and DNA sequencing analysis.

### Fluorescence Microscopy

For microscopy observation, the strains contain fluorescent fusion (s) were grown overnight on LB plates with gentamicin (50 μg/ml), then using sterile toothpicks to pick single colony into 10 μl PBS and 1 μl culture was spotted on a slide which was coated with 1% agarose. Phase contrast and fluorescence microscopy were performed by using Observer Z1 equipped with sCMOS camera (Zeiss, Germany).

### Bacterial Two-Hybrid Assays

Protein-protein interaction was detected by using the BacterioMatch II two-hybrid system (Stratagene) following the manufacturer’s protocol. Bait and prey plasmids harboring pBT-*proE* and pTRG-*proE* were co-transformed into the reporter strain XL1-Blue MRF’ Kan by electroporation. The co-transformed cells were grown on M9^+^His-deficient medium containing 5 mM 3-AT for 24–72 h at 30°C. Colonies that grew on these plates were selected as positive colonies. Then the positive colonies were subsequently picked and re-streaked on M9^+^ His-deficient plate containing 5 mM 3-AT and 12.5 mg/ml streptomycin. Normal growth on the selective screening medium indicates a positive protein-protein interaction.

### 3D Homology of ProE

The amino acid sequence for ProE (PA5295) was obtained from the *P. aeruginosa* database. Generation of the 3D homology for ProE was performed using the SWISS-MODEL workspace (Waterhouse et al., 2018). Three templates 4Y9M (PA3825-EAL-Apo), 4Y9P (PA3825-EAL-Ca^2+^, c-di-GMP), and 5M3C (PA0575-GGDEF-EAL-Ca^2+^,GTP) were selected to generate the 3D models of ProE. The molecular visualization, model analysis and image design of the ProE models, and the interactions between the wild type and mutant residues were performed using PyMOL (V. 2.3.5).

## Results

### Mutation of *proE* Results in Wrinkly Colony Morphology in *P. aeruginosa* PAO1

**FIGURE 1:**
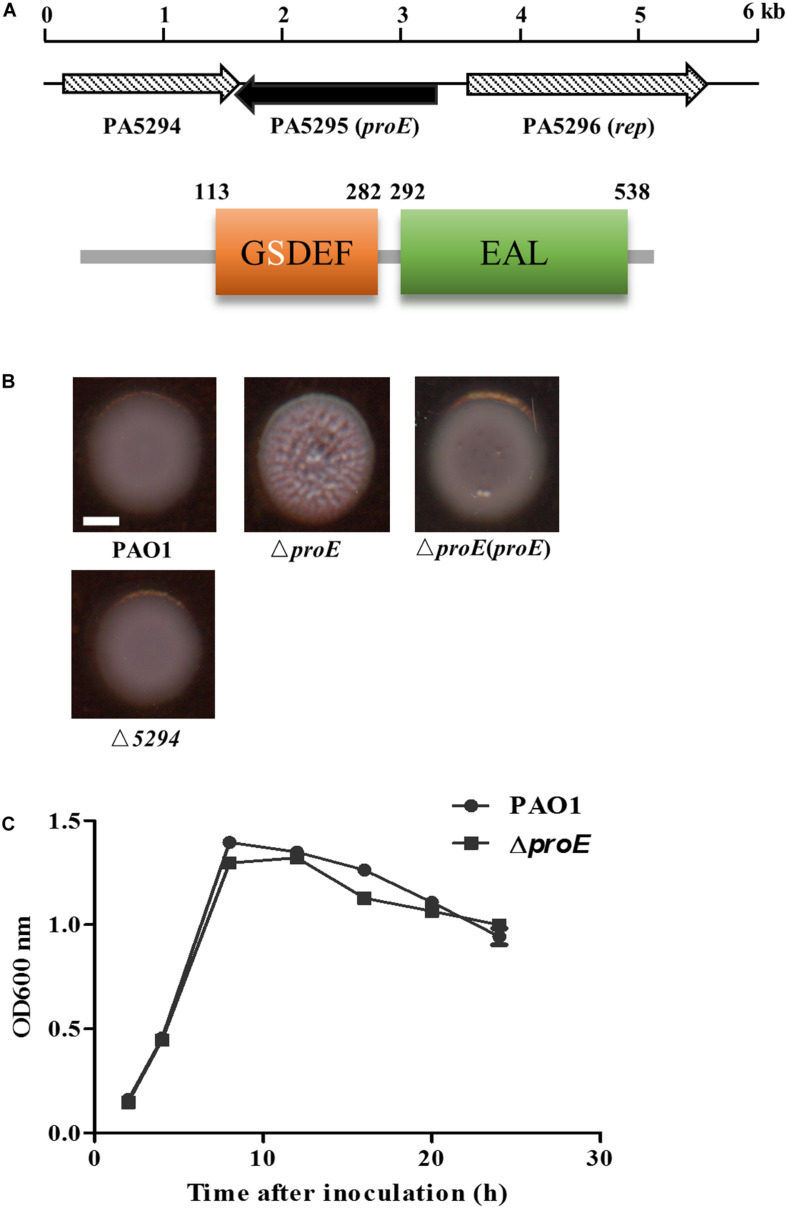
The *proE* mutant showed a wrinkly colony morphology. **(A)** Genetic organization and domain structures of ProE, and the active site of the GGDEF domain is degenerate. **(B)** Mutation of *proE* affected the colony morphology on Congo-Red plate after growth on 25°C for 2 days, nor does mutation of *PA5294* have the similar phenotype. Scale bar = 2 mm. **(C)** Growth curves of PAO1 and Δ*proE.* The data are means of three replicates and error bars indicate standard deviation.

In the process of construction of Tn5 mutant library of *P. aeruginosa* strain PAO1, we found that mutation of *PA5295*, which encodes a hypothetical protein containing a GGDEF domain and an EAL domain (Figure 1A), resulted in wrinkly colonies. A previous study by Ha et al. (2014) showed that deletion of *PA5295* resulted in increased EPS production and decreased motility in *P. aeruginosa* strain PA14, but how *PA5295* influences these phenotype changes was not determined. In addition, strains PAO1 and PA14 differ in the genes encoding EPS production, with the former containing the *psl* and *pel* gene clusters and the later containing only the *pel* gene cluster (Kuchma et al., 2007), suggesting that the scope and impact of *PA5295* in these two strains might not be the same.

To explore the function of *PA5295* in *P. aeruginosa* PAO1, we generated an in-frame deletion mutant of *PA5295*. The deletion mutant didn’t show growth defect compared with the wild type strain PAO1 (Figure 1B). Then we tested its phenotypes including motility, biofilm formation, and EPS production. We found that the *PA5295* null mutant formed colonies showing wrinkled appearance, which was different to the colony morphology of the wild-type strain PAO1 on tryptone agar plates (Figure 1B). For verification, the wild type *proE* was cloned into the expression plasmid pBBR1-MCS5, the resulting construct was introduced into Δ*proE*, which fully restored the colony morphology of Δ*proE* to the level of wild type strain PAO1 (Figure 1B).

Deletion of *proE* did not affect biofilm formation (Supplementary Figure S1), which was consistent with the findings of the previous studies (Kulasakara et al., 2006; Ha et al., 2014). However, unlike in strain PA14 (Ha et al., 2014), the *proE* null mutation did not affect bacterial motility in strain PAO1 (Supplementary Figure S1), indicating the regulatory divergence in different *P. aeruginosa* strains. Given its role in regulation of the EPS gene expression in *P. aeruginosa* PAO1, as described below, the *PA5295* was designated as *proE.*

Genome organization analysis showed that the upstream gene of *proE* is *PA5296* known as *rep*, encoding an ATP-dependent DNA helicase, and its downstream gene is *PA5294*, which encodes a putative multidrug efflux and H + -coupled pump protein belonging to the MATE family (He et al., 2004). We noticed that the ORF of *PA5294* has an 89 bp overlap with *proE* in opposite transcriptional directions. To determine whether *proE* and *PA5294* are functional associated, we constructed an *PA5294* null mutant and assayed its morphology on a Congo-Red plate, the result showed that mutation of *PA5294* did not result in obvious morphology changes compared with the wild type PAO1 (Figure 1B).

### ProE Negatively Regulates the Transcriptional Expression of *pel* and *psl*

**FIGURE 2:**
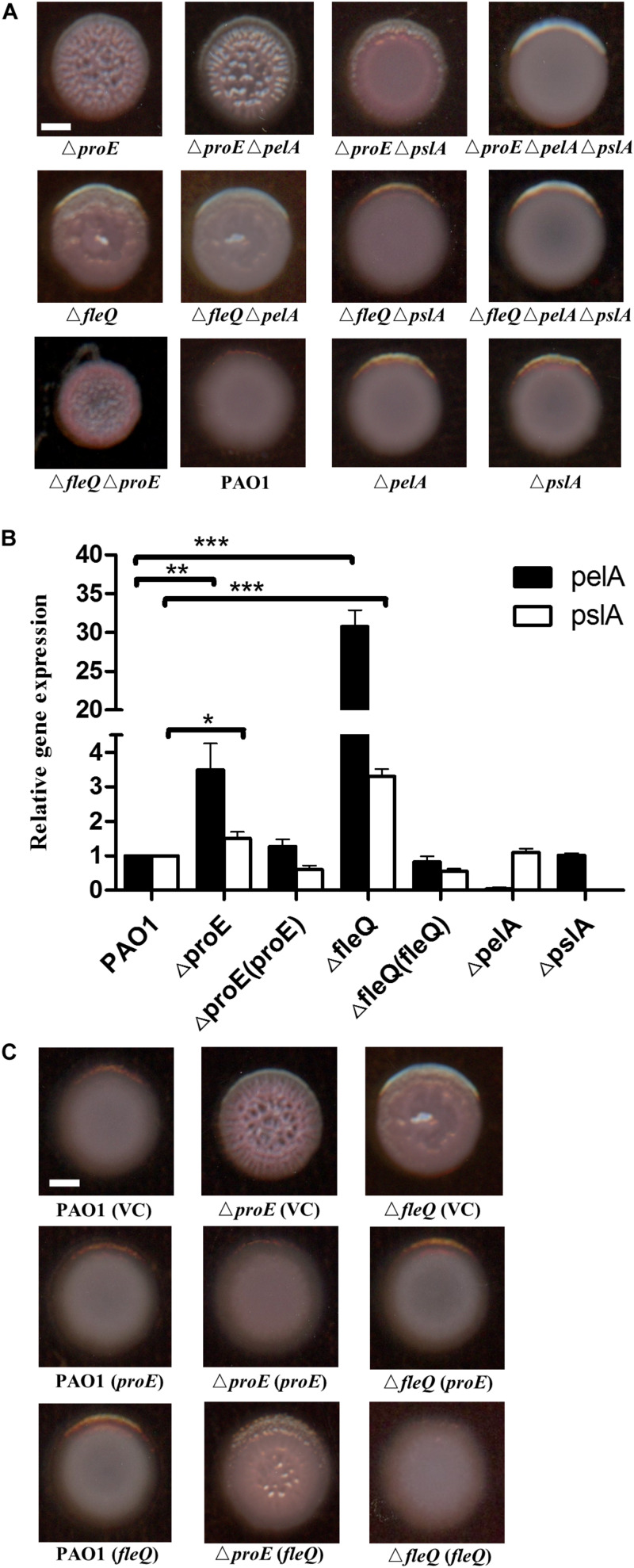
ProE regulated EPS production was dependent on *pel* and *psl*. **(A)** The wrinkly colony is only abolished when both *pelA* and *pslA* are mutated in the Δ*fleQ* background, while a *pslA* mutation is not sufficient to confer smooth colony morphology in the Δ*proE* background. All strains were growth on Congo-Red plates. **(B)** The relative gene expression of *pelA* and *pslA in* PAO1, Δ*proE*, Δ*fleQ*, Δ *proE (proE)*,Δ*fleQ (fleQ)*,Δ*pelA*, and Δ*pslA* by qRT-PCR analysis. The data are means of three replicates and error bars indicate standard deviation. *P < 0.05; **P < 0.01; ***P < 0.0001. **(C)** Complementation of *proE* can restore the wrinkly colony phenotype of Δ*fleQ*, and complementation of *fleQ* can also substantially alleviate the wrinkly colony phenotype of Δ*proE*. Scale bar = 2 mm.

Previous studies have shown that the wrinkly colony was mainly caused by increased production of Pel or Psl EPS, such as deletion of *wspF* increases the *pel* and *psl* transcription thus causing formation of wrinkly colonies (Hickman et al., 2005). In *P. aeruginosa*, the expression of *pel* and *psl* were transcriptionally regulated by the c-di-GMP binding protein FleQ, which is also the master regulator of flagella gene expression (Hickman and Harwood, 2008; Baraquet et al., 2012; Baraquet and Harwood, 2016). To use FleQ as a control, we constructed a *fleQ* null mutant, and compared the colony morphology of the mutants Δ*fleQ* and Δ*proE*. The results showed that similar to Δ*proE*, Δ*fleQ* also generated wrinkly colonies, agreeable with previous report (Hickman and Harwood, 2008). Then we generated deletions of *pelA* and *pslA* in the Δ*proE*, Δ*fleQ* and wild type background, the results showed that deletion of *pelA* in the Δ*proE* or Δ*fleQ* background significantly decreased its ability to bind to Congo-Red with relatively white colonies but wrinkly morphology remained (Figure 2A). In contrast, deletion of *pslA* in the Δ*proE* or Δ*fleQ* background did not affect Congo-Red binding but substantially reduced the wrinkly colony morphology, while it still remained partial wrinkly at edge of the colony in the Δ*proE* background (Figure 2A), and double deletion of *pelA* and *pslA* in Δ*proE* or Δ*fleQ* background fully restored the colony morphology similar to that of wild type PAO1 (Figure 2A), which clearly established the link between the wrinkly colony morphology and the increased expression of *pel* and *psl* genes in the *proE* deletion mutant. We also generated the *proE* and *fleQ* double mutant, curiously, which produced even smaller, rough and red colony appearance than the corresponding single deletion mutants (Figure 2A), suggesting a synogentics effect of two regulators on the colony morphology of *P. aeruginosa*. Consistent with this notion, we found that deletion of *proE* did not affect the transcriptional expression of *fleQ* and vice versa (Supplementary Figure S2).

To confirm the findings described above, the transcript levels of *pelA* and *pslA* in different backgrounds were determined at an optical density at 600 nm (OD_600_) about 0.5. The quantitative real-time polymerase chain reaction (qRT-PCR) results showed that the transcriptional expression of *pelA* was increased by 3.5 and 30.8 fold in Δ*proE* and Δ*fleQ*, respectively, compare with the wild type (Figure 2B). The expression of *pslA* was also increased 1.5 and 3.3 fold in Δ*proE* and Δ*fleQ*, respectively (Figure 2B). The transcript levels of *pelA* and *pslA* were higher in Δ*fleQ* than in Δ*proE*, agreeable with the role of FleQ as the downstream regulator of EPS production in *P. aeruginosa*. We also determined the gene expression of *pelA* and *pslA* in the complemented strains Δ*proE (proE)* and Δ*fleQ (fleQ)*, the result showed that transcriptional expression of *pelA* and *pslA* was decreased to the wild type level, respectively (Figure 2B).

To further explore whether *proE* and *fleQ* could functionally replace with each other, we introduced the expression constructs pBBR1-MCS5-*proE* and pBBR1-MCS5-*fleQ* into Δ*fleQ*, Δ*proE* and wild type PAO1, respectively, using empty vector as a control. The results showed that empty vector or overexpression constructs didn’t influence the colony morphology (Figure 2C). It appeared rational that *in trans* expression of *fleQ*, which encodes a c-di-GMP effector, could alleviate the wrinkly colony morphology of Δ*proE* (Figure 2C). Intriguingly, however, the wrinkly colony of Δ*fleQ* was fully rescued by complementation with *proE* (Figure 2C). Given the finding that EPS production was de-repressed after FleQ binds to c-di-GMP (Hickman and Harwood, 2008; Baraquet et al., 2012; Baraquet and Harwood, 2016), it seemed not logical that *in trans* expression of *proE*, which would decrease intracellular c-di-GMP level, could still result in reduced EPS production in Δ*fleQ.* One plausible explanation is that in addition to FleQ, there is another c-di-GMP-dependent repressor that could inhibit EPS production. Similar to FleQ, whose function in suppression of EPS production is also inactivated by c-di-GMP. Taken together, the above data showed that ProE negatively regulated EPS production by downregulating the transcriptional expression of *pel* and *psl*.

### Sequence Alignment of the GGDEF Domain and EAL Domain of ProE

**FIGURE 3:**
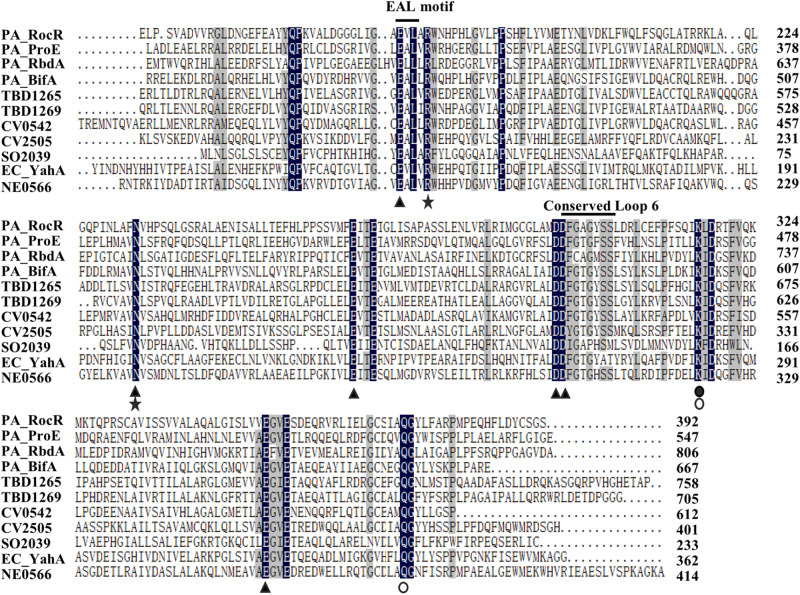
Sequence alignment of EAL-containing phosphodiesterases. The amino acid sequence of RocR (Q9HX69), ProE (Q9HTQ9), RbdA (Q9I580), BifA (Q9HW35) from *P. aeruginosa*, TBD1265 (Q3SJE6), TBD1269 (Q3SJE2) from *Thiobacillus denitrificans*, CV0542 (Q7P0M4), CV2505 (Q7NV41) from *Chromobacterium violaceum*, SO2039 (Q8EFE2) from *Shewanella oneidensis*, YahA (P21514) from *Escherichia coli*, NE0566 (Q82WU5) from *Nitrosomonas europaea.* The amino acids highlighted with black stands for 100% similarity, and gray indicates similarity level ≥75%. E^328^, N^387^, E^419^, D^449^, D^450^, E^506^ (filled triangle) are conserved residues which may bind to metal ion (Tamayo et al., 2005; Rao et al., 2008; Tchigvintsev et al., 2010; Yang et al., 2017). The conserved loop 6 (DFG(T/A)GYSS) is essential for the dimerization of EAL domain and Mg^2+^, c-di-GMP binding (Rao et al., 2009). R^332^, N^387^ are (asterisks) reported to bind with substrate (Rao et al., 2008; Tchigvintsev et al., 2010; Yang et al., 2017). K^470^ (filled circle) is essential for coordinating with water molecule for catalysis and interacting with residue E^419^ residue (Rao et al., 2008; Tchigvintsev et al., 2010; Yang et al., 2017). Q^526^ is reported to interact with E^328^ and K^470^ (open circle) (Tchigvintsev et al., 2010; Yang et al., 2017).

ProE is a hybrid protein with a GGDEF domain (113–282 aa) and an EAL domain (292–538 aa), which are known to be involved in c-di-GMP biosynthesis and degradation, respectively. To understand whether these two domains are well conserved, we firstly compared the amino acid sequence of GGDEF domain of ProE with the functionally characterized DGCs. We found that the ProE contains most conserved residues, however, three key residues, including c-di-GMP binding site (E^192^), GG(D/E)EF signature motif (S^202^), and GTP binding site (R^276^) are mutated (Supplementary Figure S3), suggesting ProE is likely to be a degenerated DGC. Then we compared the amino acid sequence of EAL domain of ProE with other functional characterized PDEs. Previous studies found that several key residues are critical for the EAL domain to perform its PDE activity (Rao et al., 2008, 2009; Tchigvintsev et al., 2010; Yang et al., 2017). We thus asked whether these key residues are also conserved in the EAL domain of ProE, we found that the most essential residues for PDE structure and activity are conserved, except that the conserved loop 6 [DDFG(A/T)GYSS] has a mutation with Y455 being changed to F455 (_449_DDFGTGFSS_457_) (Figure 3). Phylogenetic tree analysis showed that ProE was quite conserved within the *Pseudomonas* genus (Supplementary Figure S4). And based on our bioinformatic analysis by using the SMART program^1^ and *Pseudomonas* website^2^, ProE lacks N-terminal transmembrane or signal sensing domain, which suggests that ProE is likely to be a soluble cytoplasmic protein, and may not directly interact with signal(s).

### ProE Is an Active Phosphodiesterase

To determine the enzyme activity of ProE, we expressed and purified the recombinant ProE, we also purified the previously functionally characterized DGC WspR and PDE RocR as positive controls (Hickman et al., 2005; Rao et al., 2008; Supplementary Figure S5). To test DGC activity of ProE, we incubated ProE with GTP for 2 h, then analyzed the products in the reaction mixture by HPLC. The result showed that while the control protein WspR could convert most GTP into c-di-GMP, ProE could not synthesis c-di-GMP (Supplementary Figure S6), suggesting that in agreement with sequence alignment analysis, ProE is not a functional DGC.

**FIGURE 4:**
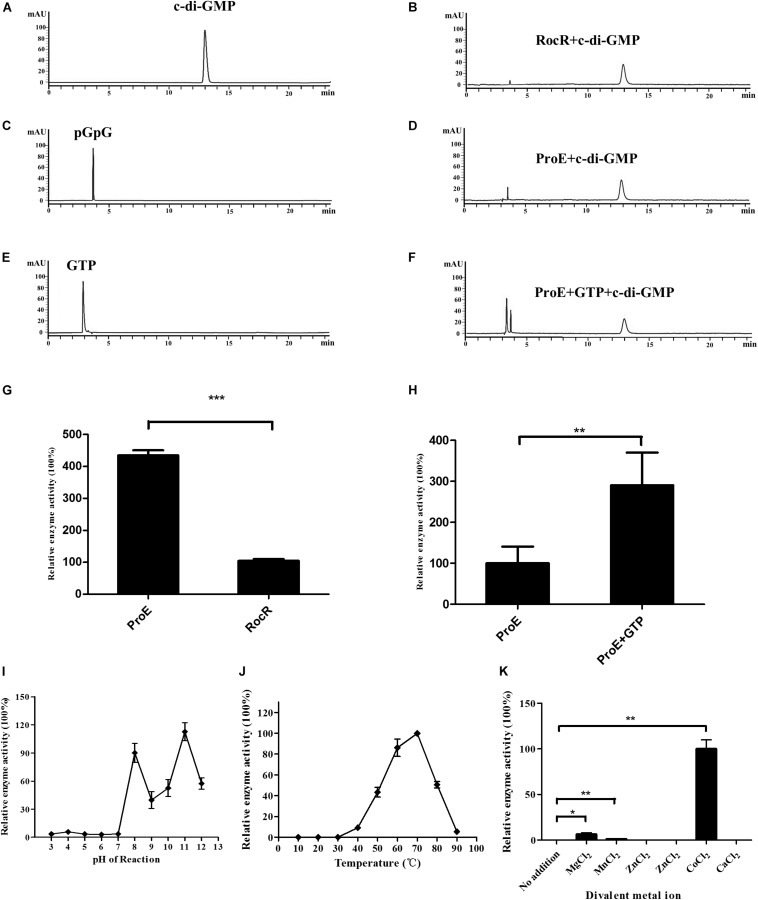
Purified ProE is an active phosphodiesterase. **(A,C,E)** The standard of c-di-GMP **(A)**, pGpG **(C)**, GTP **(E)** in a final concentration of 100 μM was prepared in the reaction buffer, and 10 μl was injected for HPLC analysis. **(B)** RocR and **(D)** ProE after incubation with c-di-GMP at a final concentration of 50 μM at 37°C for 20 min. **(F)** ProE incubated with 50 μM GTP for 5 min at room temperature prior to addition of c-di-GMP, then the enzyme activity was analysis by HPLC. **(G)** Quantification of the enzyme activity of ProE and RocR. **(H)** Quantification of the enzyme activity of ProE and ProE with GTP prior to addition substrate. **(I)** The influence of pH on enzyme activity. The pH of the phosphodiesterase reaction was adjusted to 3–12 prior to the addition of enzyme and substrate (37°C, 5 mM MgCl_2_). ProE activity was higher in alkaline condition. **(J)** The influence of temperature on enzyme activity. The ProE phosphodiesterase reaction was performed at a range of temperatures from 10°C to 90°C (pH 8.0, 5 mM MgCl_2_). The highest rate of c-di-GMP hydrolysis was achieved at 60°C and 70°C. At temperatures lower than 30°C, no activity was detectable. **(K)** Dependence of the phosphodiesterase activity of ProE on divalent metal cations. Reaction buffer (37°C, pH 8.0) The data are means of three replicates and error bars indicate standard deviation. *P < 0.05; **P < 0.01, ***P < 0.0001.

Then we tested the PDE activity of ProE, after incubation with c-di-GMP at 37°C for 20 min, the reaction was stopped. HPLC analysis showed that the degradation product pGpG was produced with a retention time at 3.7 min, indicating that ProE is an active PDE (Figure 4D). Interestingly, we noticed that ProE was more active than RocR (Figures 4B,D). Quantitative analysis showed that the enzyme activity of ProE was about 4 folds higher than RocR (Figure 4G), which was shown to be a highly active PDE (Kulasakara et al., 2006).

It was reported that GTP can allosterically control PDE activity (Christen et al., 2005). We then measured the PDE activity of ProE in the presence or absence of GTP under the same reaction conditions. Similar to other GGDEF-EAL fusion domain proteins (Christen et al., 2005; An et al., 2010; Bellini et al., 2017; Mantoni et al., 2018), the PDE activity of ProE was greatly enhanced by about 2.9 folds after supplementation with 50 μM GTP in the reaction mix prior to addition of c-di-GMP (Figures 4D,F,H).

Previous studies showed that several PDEs are active homodimers (Barends et al., 2009; Minasov et al., 2009; Robert-Paganin et al., 2012; Sundriyal et al., 2014; Bellini et al., 2017; Mantoni et al., 2018), whereas RocR has a unusual tetrameric structure (Chen et al., 2012),the dimerization of EAL domain is critical for the function PDEs (Rao et al., 2009; Bellini et al., 2017; Mantoni et al., 2018). And the GGDEF domain also plays important role as a scaffold to ensure such dimerization (Mantoni et al., 2018). By using bacterial two-hybrid approach, we found that ProE could interact with ProE (Supplementary Figure S7), implying that ProE is also likely to exist as an oligomer.

To test the substrate specificity of ProE, we added other three nucleotide second messengers including cAMP, cGMP, and c-di-AMP into the reaction mix and incubated at 37°C for 20 min prior to HPLC analysis. The results showed that ProE could only degrade c-di-GMP, but not cAMP, cGMP, or c-di-AMP (Supplementary Figure S8), indicating that ProE is a c-di-GMP specific PDE.

### Analysis of the Optimal ProE Reaction Conditions

To investigate the optimal reaction conditions for the PDE activity of ProE, we tested the effect of various environmental factors including pH, temperature and divalent cations on ProE activity. The results showed that ProE displayed a low enzyme activity from pH 3 to 7, and enzyme activity was substantially increased at pH ranging from 8 to 11 (Figure 4I). Enzyme activity of ProE was also determined at temperature ranging from 10°C to 90°C, and ProE showed a very low activity when the temperature was lower than 30°C with the maximum enzyme activity being at 70°C (Figure 4J). PDE enzymes commonly need divalent cation for their enzyme activity. Several cations, including Mg^2+^, Mn^2+^, and Co^2+^ could significantly boost the enzyme activity of PDEs, whereas Ca^2+^, Fe^2+^, Ni^2+^, Zn^2+^ don’t support the PDE activity (Bobrov et al., 2005; Schmidt et al., 2005; Tamayo et al., 2005; Barends et al., 2009; Tchigvintsev et al., 2010). We test the ProE activity by addition of above divalent cations to the reaction buffer, respectively. Similar to other known PDEs, the cations Ca^2+^, Fe^2+^, Ni^2+^, Zn^2+^ didn’t support the enzyme activity of ProE (Figure 4K). However, unlike other PDEs, which showed the highest enzyme activity in the presence of Mn^2+^ or Mg^2+^ (Bobrov et al., 2005; Schmidt et al., 2005; Tamayo et al., 2005; Barends et al., 2009; Tchigvintsev et al., 2010), Co^2+^ was shown to be the best metal ion for the enzyme activity of ProE (Figure 4K).

### Identification Three Novel Residues Critical for ProE Activity

**FIGURE 5:**
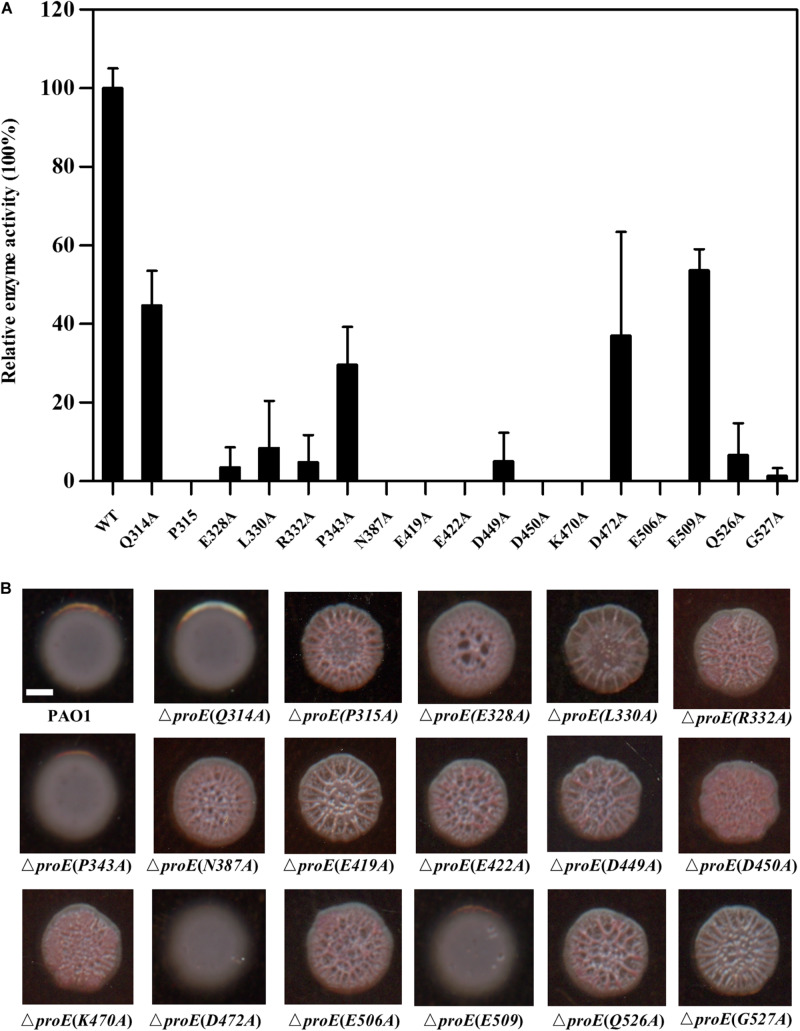
Functional analysis of the conserved residues of EAL domain of ProE. **(A)** After site-directed mutagenesis, the enzyme activity of mutant proteins were analyzed. The data are means of three replicates and error bars indicate standard deviation. **(B)** Effect of conserved residues on the colony morphology of *P. aeruginosa* strain PAO1 and its derivatives on Congo-Red plates. The native *proE* gene is deleted, and the *proE* alleles are according to complementation of mutated pBBR1-MCS5-*proE* into Δ*proE.* Scale bar = 2 mm.

According to the amino acid sequence alignment, we identified a total of seventeen conserved residues in the EAL domains of the functional PDEs (Figure 3). To verify the roles of these conserved residues in the hydrolysis of c-di-GMP, we mutated these residues to alanine (Ala, A) and assayed the activity of purified mutant proteins. Among these proteins, four proteins showed a decreased enzyme activity (less than 55% of the wild type enzyme) (Q314, PA343, D472, E509) with unknown functions, nine proteins with mutations in the metal-binding residues showed an extremely low or abolished enzyme activity (E328, R332, N387, E419, E422, D449, D450, K470, E506, Q526), agreeable with the above observation that metal ions played a critical role in ProE catalysis (Figure 5A and Supplementary Table S3). Three proteins with mutations in the residues involved in substrate binding or coordination with water molecule (R332, N387, K470), as well as two proteins with mutations in the residues involved in interaction with other residues (K470, Q526) also showed compromised enzyme activity (Figure 5A and Supplementary Table S3). Significantly, this study unveiled three novel residues which were critical for ProE activity (P315, L330, G527) (Figure 5A and Supplementary Table S3). The proteins with mutation in these residues showed a very low enzyme activity (less than 10% of the wild type enzyme), indicating their indispensable roles in catalysis. Their functional roles in catalysis remain to be further characterized.

We wanted to know whether the above key residues are also important for the ProE function *in vivo*. To address this question, we introduced the *proE* derivatives into Δ*proE* and assayed their colony morphology by Congo-Red plate. The results showed that substitution of thirteen residues with Ala failed to restore the colony morphology of Δ*proE*, including P315, L330, E328, R332, N387, E419, E422, D449, D450, K470, E506, Q526, and G527 (Figure 5B), which was consistent with our *in vitro* data, and confirmed the important roles of these residues for ProE function. The remaining four ProE derivatives (Q314, P343, D472, E509) could restore the Δ*proE* colony morphology to the wild type PAO1 level (Figure 5B). Our *in vitro* enzyme assay, showed that these ProE derivatives could still degrade c-di-GMP with activity about 29.6–53.7% of the wild type ProE. It is likely that these derivatives were overexpressed due to the multi-copy nature of the complementary expression vector, thus keeping the concentration of intracellular c-di-GMP to a level similar to wild type PAO1.

### ProE EAL Domain 3D Model Comparison and Analysis

**FIGURE 6:**
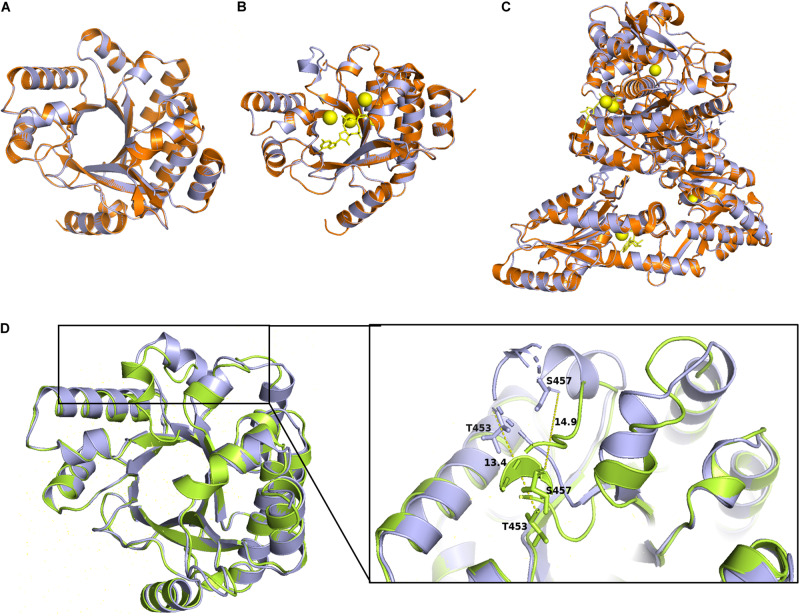
Homology models of the ProE with PA3825 and RmcA. Superimposition of the wild type ProE models with templates of 4y9m (PA3825-Apo), RMSD = 0.064 (214 to 214 atoms) **(A)**, and 4y9p (PA3825-Ca^2+^ and c-di-GMP), RMSD = 0.04 (213 to 213 atoms) **(B)**, and 5m3C (RmcA-Ca^2+^-GTP), RMSD = 0.190 (721 to 721 atoms) **(C)**. ProE and templates are shown in lightblue and orange cartoons, respectively. C-di-GMP and GTP are shown as yellow sticks, calcium ions are shown in yellow spheres. **(D)** Superposition of Apo-ProE and ligand-bound ProE models reveals conformational changes due to ligand interaction. Apo-ProE and ligand-bound ProE are shown in lightblue and lime cartoons, respectively. The number represents the distance between residues (Å).

To further analysis the role of the identified conserved residues, especially the newly identified three residues, i.e., P315, L330, and G527. We use homology modeling approach to computationally generate structural models based on two previously characterized PDEs PA3825 and RmcA (Bellini et al., 2017; Mantoni et al., 2018). The Apo and the ligand-bound (Ca^2+^ and c-di-GMP) templates from PA3825 were used to generate two different structures, whereas the other models were obtained from the ligand-bound (Calcium and GTP) RmcA. The superposed model of the ProE with the x-ray structures of PA3825 and RmcA revealed very high similarities in the conformation of the EAL domain (RMSD < 1) (Figures 6A–C). The PA3825 Apo and PA3825 (Ca^2+^, c-di-GMP) models showed low structural differences when superposed on the ProE model with RMSD of 0.064 Å and 0.045 Å, respectively, for 214 atoms (Figures 6A,B). Superposition of the x-ray structure model of RmcA-GGDEF-EAL-Ca^2+^GTP with the ProE model also exhibited a high structural similarity (0.190 Å for 721 atoms) (Figure 6C). As expected, the superposed structures of the Apo-ProE and ligand-bound ProE models demonstrated a significant variation in conformation around the alpha 5 helix, which was reported to enable catalytic metal interaction and dimerization (D160 and D161 in PA3825: D449 and D450 in ProE) (Rao et al., 2009; Bellini et al., 2017; Figure 6D).

**FIGURE 7:**
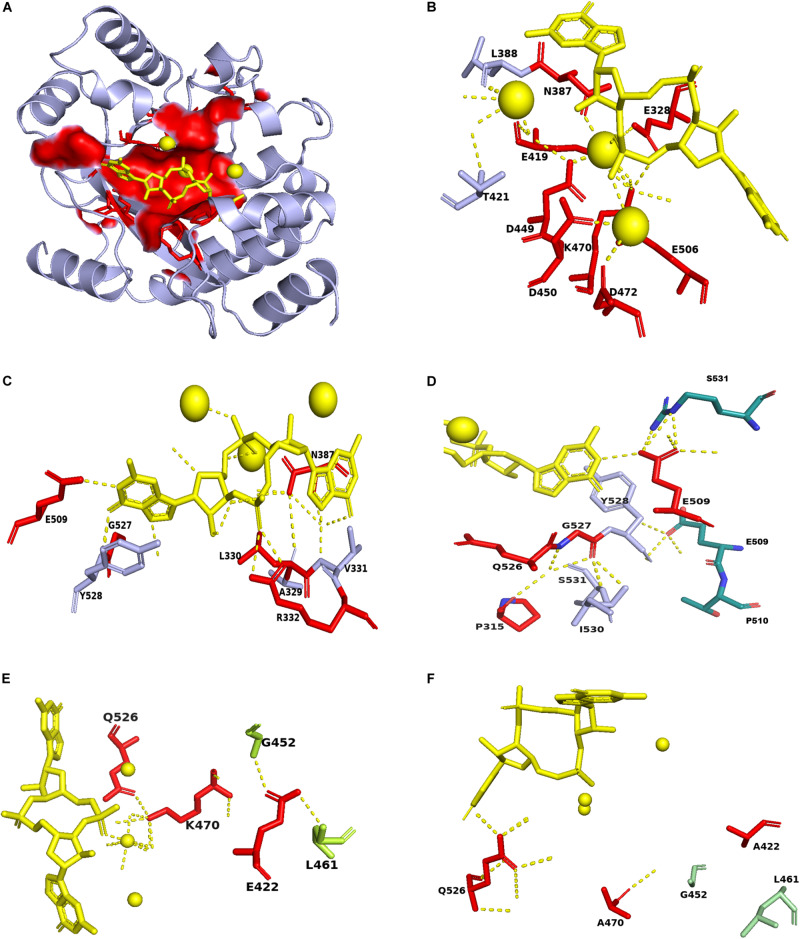
Mapping of conserved residues identified in ProE with homolog model. **(A)** The surface structure showing the localization of the conserved residues in the ProE model around the calcium ions and c-di-GMP. **(B)** The labeled residues indicate those interact with the calcium ions in ProE. **(C)** The labeled residues indicate those form direct or indirect polar contacts with c-di-GMP. **(D)** The residues E509 and Y528 interact with S531 and E509, respectively, from another monomer to allow dimer formation. **(E)** Interaction of conserved residues K470 and E422 with their adjacent residues. **(F)** Interaction of K470A and E422A with their adjacent residues. Calcium ions and c-di-GMP molecules are shown as yellow spheres and sticks, respectively. Polar interaction are shown as yellow dashes.

Then we map the identified conserved residues in ProE with the PA3825-Apo, PA3825(Ca^2+^, c-di-GMP), RmcA-GGDEF-EAL-Ca^2+^GTP templates, the result showed that the most residues are distributed in the ligand binding region (Figure 7A). Superposition of the conserved residues of ProE model with PA3825(Ca^2+^, c-di-GMP), showed that the residues E328, N387, E419, D449, D450, D472, and E506 enable catalytic metal ion (Ca^2+^) interaction (Figure 7B). The residues R332, N387, E509 interact with the c-di-GMP molecule (Figure 7C). Additionally, from the c-di-GMP bound ProE model, E509 is located in the dimerization interface and enables the formation of dimers between the two monomers (Figure 7D). The residue E422 forms polar interaction with L461 and G452, whiles K470 forms a polar interaction with Q526 and a water-mediated hydrogen bond with the calcium ion (Figure 7E). Polar interactions of E422 with L461 and G452, respectively, were affected by the E422A mutation (Figure 7F). Likewise, the water-mediated hydrogen bonds between K470, calcium ion and c-di-GMP were affected by the K470A mutation (Figure 7F).

Next, we investigated the potential roles in catalysis of the three newly identified residues P315, L330, and G527 using the model generated from PA3825(Ca^2+^, c-di-GMP). We observed a water-mediated hydrogen bond interaction between the residue P315 and G527 (Supplementary Figure S9A), however, this interaction was not abolished in both P315A and G527A mutants (Supplementary Figures S9B,C). Substitution of P315 with A315 did not affect the interaction of the neighboring residues, i.e., Q314 with S531, and Q526 with E328 (Supplementary Figures S9D,E). No conformational changes were observed when the wild type ProE and P315A-ProE mutant models were superposed with the two ligand-bound PA3825 templates (RMSD = 0, 212 atoms) (Supplementary Figure S9F).

From the ProE model generated with the PA3825 (Ca^2+^c-di-GMP) template, L330A mutation did not seem to affect the polar interaction between L330 and H312, nor did it affect the neighboring residues E328, A329, V331, and R332 (Supplementary Figures S10A,B). Also, the mutation L330A did not result in a conformational change when superposed with the wild type ProE model generated with 4Y9P (RMSD = 0, 227 atoms) (Supplementary Figure S10C).

The dipeptide bond between G527 and Y528 enables interaction with the c-di-GMP molecule. It is unclear how this G527 influences binding of c-di-GMP as G527A mutation did not seem to affect c-di-GMP polar interaction as well as the interaction mediated by adjacent residues (Supplementary Figures S10D,E). Substitution of the adjacent residue Q526 with A526, however, resulted in loss of polar interaction between A505 and K470 (Supplementary Figure S10E).

According to above analysis we re-confirm the role of residues involved in substrate binding (R332 and N387), metal ion binding (E328, N387, E419, D449, D450, and E506), and residue-residue interaction (K470 and Q526) (Figure 7 and Supplementary Figures S9, S10, Supplementary Table S3). We found that the residues in which mutation resulted in decreased enzyme activity in our study and previous study with unknown functions (Supplementary Table S3), such as E422, D472, and E509, may play role in residue-residue interaction, metal ion binding and substrate binding, respectively (Figure 7 and Supplementary Figure S9). As for the newly identified three residues P315, L330, and G527, homolog model analysis seem to preclude their involvement in substrate or metal-ion binding (Figure 7 and Supplementary Figures S9, S10). How these residues affect the enzyme activity of ProE remain to be further investigated.

### EPS Production Was Synergistically Regulated by Several PDEs in *P. aeruginosa* PAO1

**FIGURE 8:**
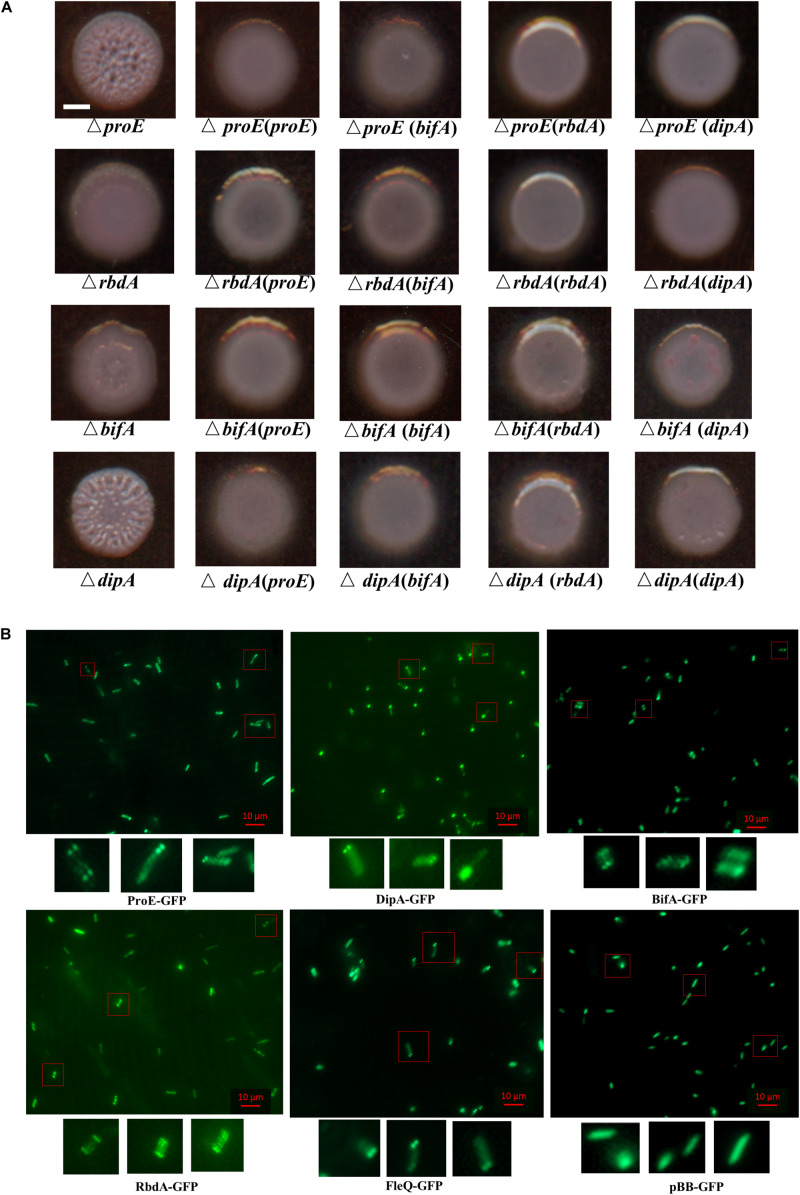
Functional and subcellular localization analysis of EPS-associated phosphodiesterases. **(A)** ProE and other three phosphodiesterases synergically regulated the colony morphology in *P. aeruginosa*. Deletion of either *proE*, *rbdA*, *bifA* or *dipA* can cause wrinkly morphology at different extent, and the functional defect can be restored by each other. Scale bar = 2 mm. **(B)** Subcellular localization of GFP fusion proteins. Bacterial colonies expressing indicated GFP fusion proteins were picked from overnight LB agar plates, resuspended in PBS and 2 μl culture was spotted onto a glass slide coated with 1% agarose. Images were taken by using epifluorescent microscopy. Scale bar = 10 μM.

In previous work, *rbdA* was shown to modulate biofilm dispersal by negatively regulating EPS production and positively regulating bacterial motility in our lab and other groups (An et al., 2010; Liu et al., 2018). Roy et al. (2012) reported that mutation of *dipA* (another name *pch*) can cause increased Congo-Red binding, suggesting its role in EPS production. In PA14 strain, *bifA* was demonstrated to inversely regulate biofilm formation and swarming motility, and the regulation on biofilm formation was dependent on *pel* (Kuchma et al., 2007). To assess the roles and compare the impact of these PDEs in regulating EPS production in *P. aeruginosa* PAO1, we generated the deletion mutants of *rbdA*, *bifA*, and *dipA*, respectively. Then we tested the effect of mutation on EPS production by Congo-Red plate assay. The results indicated that mutation of *rbdA*, *bifA*, and *dipA*, respectively, resulted in enhanced Congo-red binding, with colonies showing different extents of wrinkles. Among them, Δ*rbdA* and Δ*bifA* showed a rough appearance, and Δ*dipA* produced wrinkly colonies like Δ*proE* (Figure 8A). The results suggest that although they are all involved c-di-GMP metabolism and EPS production, the extent of their regulation may not be exactly the same. We were curious whether these PDEs could functionally replace each other. The results showed that *proE*, *rbdA*, *bifA*, and *dipA* could functionally restore the colony morphology of each other to the wild type PAO1 level (Figure 8A). These data support the notion that *P. aeruginosa* might use different PDEs for subtle control of c-di-GMP homeostasis and EPS production.

### Subcellular Localization of EPS Associated PDEs and Master Regulator FleQ

Previous studies showed that a set of proteins involved in c-di-GMP metabolism share a distinct subcellular localization in *P. aeruginosa* and other bacterial species (Guvener and Harwood, 2007; Yang et al., 2012; Kulasekara et al., 2013; Valentini et al., 2016; Jain et al., 2017; Xue et al., 2018; Li et al., 2019). We wanted to know whether the EPS associated proteins ProE, RbdA, BifA, DipA, and FleQ share a link between their function and subcellular localization. To address this question, we constructed C-terminal GFP fusions of ProE, RbdA, BifA, DipA, and FleQ, respectively, then their subcellular localizations were observed in *P. aeruginosa* PAO1 cells under fluorescence microscopy (Figure 8B). As shown in Figure 8B, DipA showed polar localization in most cells (more than 50%), which was consistent with previous study (Kulasekara et al., 2013). ProE, BifA, and FleQ showed mostly polar localization, including unipolar localization, bipolar localization. RbdA showed less polar localization, and a fraction of cells showed multiple-point distributions. As control, we also introduced the empty GFP into PAO1, the GFP proteins are uniformly distributed in the cells (Figure 8B). The above findings seem to suggest a general pattern of polar localization of these EPS-associated regulators, which may be important for precise regulation of EPS production in bacteria cells.

## Discussion

In this study, we showed that ProE played a key role in regulating EPS production in *P. aeruginosa* strain PAO1 through modulation of the transcriptional expression of the *pel* and *psl* gene clusters (Figures 1, 2). We further demonstrated that ProE was a highly active PDE, whose maximum activity required the cation Co^2+^ (Figure 4). Furthermore, by scanning mutagenesis, we identified three novel key residues for the function of ProE *in vitro* and *in vivo* (Figure 5 and Supplementary Table S3), and these three residues are conserved in other functional PDEs. Moreover, we showed that several PDEs, including RbdA, BifA, DipA, and ProE, could functionally replace each other in regulation of the EPS-related colony morphology, and these enzymes, together with the EPS regulator FleQ, shared a general pattern of polar localization, which may suggest a link between localization and functionality in regulation of EPS production (Figure 8). These findings expand our understandings on the enzymatic properties of c-di-GMP metabolic enzymes, and provide a new insight on the regulatory mechanisms of c-di-GMP metabolic proteins on EPS production and colony morphology.

The *proE* gene was identified as its mutation led to formation of wrinkly colonies. Previous studies showed that wrinkly colony morphology is caused by enhanced EPS production (Hickman et al., 2005; Hickman and Harwood, 2008). Unlike other functionally characterized PDEs that commonly influence biofilm formation and motility (Kuchma et al., 2007; An et al., 2010; Yi et al., 2010; Deng et al., 2012; Roy et al., 2012; Chao et al., 2013; Rossello et al., 2017). The function of ProE seems to be specific to EPS production. Deletion of proE resulted in significantly elevated transcriptional expression of the *pel* and *psl* genes responsible for EPS production, but did not influence other phenotypes like biofilm formation or motility (Figure 1 and Supplementary Figure S1). And interestingly, we noticed that ProE showed some similarities with PA3177, which controls the metabolism of intracellular c-di-GMP without influences biofilm formation. Given that ProE plays a key role in regulating EPS production, which are critical for antibiotics resistance (Murakami et al., 2017), it is possible that ProE may function like PA3177 by regulating the biofilm-mediated antibiotics resistance (Poudyal and Sauer, 2018). The phylogenetic analysis showed that ProE is conserved in other *Pseudomonas* species as well, including *Pseudomonas syringae pv. tomato* DC3000, *Pseudomonas savastanoi pv. phaseolicola* 1448A, *Pseudomonas putida* F1 and *Pseudomonas fluorescens* SBW25 (Supplementary Figure S4), suggesting that ProE is a widely conserved c-di-GMP metabolic enzyme. It would be interesting to determine whether ProE may also play a role in regulating EPS production and colony morphology in these bacterial species. Prior to this study, the biological functions of ProE has only been investigated in *P. aeruginosa* strain PA14, in which ProE was shown to play a role in regulation of EPS biosynthesis and bacterial motility (Ha et al., 2014). In contrast, the ProE of strain PAO1, which shares the same amino acid sequences with its counterpart in strain PA14, only regulated EPS biosynthesis without affecting bacterial motility. These findings may suggest that the functional divergence of ProE occurred in the process of bacterial evolution. The underpinning regulatory mechanisms of functional divergence await further investigations.

In previous studies, several groups used *proE* for heterogenous complementation, which could rescue the c-di-GMP dependent phenotypes (Malone et al., 2010, 2012; Davis et al., 2013; Skotnicka et al., 2016a, b; Schmid et al., 2017; Skotnicka and Sogaard-Andersen, 2017; Berne et al., 2018; Laventie et al., 2019), suggesting its PDE activity *in vivo*. However, the enzyme properties of ProE have not yet been characterized in detail. Our data showed that ProE was a very active PDE with catalytic activity much higher than RocR (Figures 4B,D,G), which was shown to be one of the most active enzymes among the *P. aeruginosa* PDEs (Kulasakara et al., 2006). It is not clear yet what features account for the high catalytic activity of ProE, but several clues may be worthy of further consideration. Firstly, compared with another highly active PDE RocR, these two highly active enzymes differs in domain structures with RocR has an extra REC domain at N-terminal, whereas ProE contains only the GGDEF and EAL domains. It was believed that under certain conditions, the REC domain of RocR could be phosphorylated, which might induce the enzyme structural changes and present the EAL domain at an active status (Chen et al., 2012). In contrast, ProE lacks the N-terminal REC domain and thus its EAL domain is likely keeping at ready and active status, and ProE has a GTP binding domain that may further increase its enzyme activity. These may explain the observed ultra high activity of ProE in comparison with RocR. Second, ProE showed the maximum enzyme activity in the presence of Co^2+^ (Figure 4K), rather than Mg^2+^ or Mn^2+^ commonly used by others PDEs (Bobrov et al., 2005; Schmidt et al., 2005; Tamayo et al., 2005; Barends et al., 2009; Tchigvintsev et al., 2010). It has been well documented that different cations may generate variable impact on enzyme structure and activity. Similarly, it was shown recently that another two PDEs, CnpB from *Mycobacterium tuberculosis* and *Vc*EAL (VC0395_A1247) from *Vibrio cholerae* also showed the best performance with Co^2+^ (Yang et al., 2014; Yadav et al., 2019). It is likely that ProE may share a similar metal-ion-dependent catalysis mechanism with these enzymes. Nevertheless, cation ion seems only affect catalysis but doesn’t influence the enzyme substrate specificity. ProE degraded only c-di-GMP (Supplementary Figure S8), whereas CnpB and *Vc*EAL could degrade not only c-di-GMP, but also c-di-AMP or cGAMP, respectively (Yang et al., 2014; Yadav et al., 2019).

By using bioinformatics analysis and site-directed mutagenesis, we found a total of seventeen residues important for the function of ProE (Figure 5). While most of these residues are previously identified and known to be involved in metal-ion and substrate binding, water molecule coordination, residue-residue interaction or dimerization (Supplementary Table S3), we also identified three novel residues, i.e., P315, L330, and G527, which are also critical for the function of ProE. These three residues are well conserved in other functional PDEs, suggesting their potential roles in these PDEs (Figure 3), but the role of these residues remains unknown, it seems that they may not play a role in substrate or metal-ion binding by our homolog model analysis (Figures 6, 7 and Supplementary Figures S9, S10). Further structural and biochemical studies will gain more insights into the detailed mechanisms of these residues in ProE.

Deletion of the four PDEs known for c-di-GMP degradation, including ProE, RbdA, DipA, and BifA, respectively, caused colony morphological changes and the mutant phenotypes could be rescued by any of these four PDEs (Figure 8A), suggesting that they are all involved in regulation of EPS production in strain PAO1. This can be interpreted that over-expression of these PDEs dramatically decreased the concentration of intracellular c-di-GMP, which frees and enables FleQ to interact with the promoter of *pel* and *psl*, thus decreasing the expression of *pel* and *psl* (Baraquet et al., 2012; Baraquet and Harwood, 2016), and rescued the EPS over-production phenotype. In previous study, several DGCs and PDEs have been found to show polar localization including SadC (Merritt et al., 2010; Zhu et al., 2016), DipA (Kulasekara et al., 2013), WspR (Guvener and Harwood, 2007), HsbD (Valentini et al., 2016). By using C-terminal tagged fusion proteins to explore their subcellular localization, we found that DipA showed polar localization (Figure 8B), which agreed with the previous study (Kulasekara et al., 2013). Intriguingly, ProE, RbdA, and BifA, as well as then EPS regulator FleQ, also showed polar localization with varied extent or percentages (Figure 8B). In particular, ProE and DipA, whose null mutants showing more obvious wrinkly phenotype that the other two PDEs (Figure 8A), showed higher degree of polar localization (Figure 8B). These evidences seem to suggest that there is a link between EPS production and cell pole localization. It is possible that somehow EPS production was regulated at cell pole when the local c-di-GMP concentration reach a threshold. The underlying mechanism remains to be further studied by *in situ* exploration of the local c-di-GMP concentration and EPS production.

In summary, the results from this study demonstrated that ProE is an highly active PDE, which plays a key role in modulation of transcriptional expression of the *pel* and *psl* genes encoding EPS production in *P. aeruginosa*. The work also presents useful clues or questions for further investigations. For example, what are the precise roles of the three newly identified key residues, i.e., P315, L330, and G527, which are highly conserved in other PDEs. It is highly intriguing why ProE affects only EPS production, whereas other PDEs such as RbdA could affect multiple phenotypes. We reported previously that RbdA plays a key role in regulation of EPS production, bacterial motility, and biofilm formation (An et al., 2010). Given that EPS production is not only regulated by c-di-GMP but also controlled by quorum sensing (He et al., 2006), it is equally thought-provoking how *P. aeruginosa* could tap to multiple signal inputs or regulatory mechanisms in modulation of EPS production, and whether and if yes, how different regulation systems could interact or interplay with each other? The findings from this study lay down a footstone for probing these challenges.

## Data Availability Statement

The datasets presented in this study can be found in online repositories, the amino acid sequences of ProE and its homolog in phylogenetic analysis are accessible in NCBI under accession numbers NP_253982.1, WP_034025707.1, WP_033966831.1, WP_033998527.1, WP_033969843.1, WP_073636980, PAZ 22036.1, WP_142901090.1, WP_031673977.1, WP_096084263.1, YP_793769.1, YP_002443268, WP_012077917.1, NP_789973.1, WP_015886513.1, WP_011064201.1, WP_003253539.1, WP_01 1167318.1, WP_011911732.1, WP_003206833.1, WP_0286836 56.1, and AAY49330.1.

## Author Contributions

L-HZ and QF conceived and designed the experiments. QF, SA, TZ, ZL, QL, YL, and JH performed the experiments. QF, SA, and JZ analyzed the data. QF and L-HZ wrote the manuscript. All authors read and approved the final manuscript.

## Conflict of Interest

The authors declare that the research was conducted in the absence of any commercial or financial relationships that could be construed as a potential conflict of interest.

## Footnotes

1 http://smart.emblheidelberg.de/

2 http://www.pseudomonas.com
