# Supplementary material for: Regulation of Exopolysaccharide Production by ProE, a Cyclic-Di-GMP Phosphodiesterase in Pseudomonas aeruginosa PAO1
Source: Front Microbiol. 2020 Jun 5;11:1226. doi: 10.3389/fmicb.2020.01226 (PMC7290235; doi:10.3389/fmicb.2020.01226)
Supplement: Supplementary file 1 [file Data_Sheet_1.docx]

**Supplementary Information**

**Content:**

**Supplementary Table S1**. Bacterial strains and plasmids used in this study.

**Supplementary Table S2.** Primers used in this study.

**Supplementary Table S3.** Analysis of the enzyme activity of ProE and its variant.

**Supplementary Figure S1.** ProE doesn’t regulate motility and biofilm formation**.**

**Supplementary Figure S2.** *proE* and *fleQ* are not transcriptionally regulated by each other.

**Supplementary Figure S3.** Sequence alignment of ProE GGDEF domain with other GGDEF-containing diguanylate cyclase.

**Supplementary Figure S4.** Phylogenetic analysis of ProE proteins from several *Pseudomonas* strains.

**Supplementary Figure S5.** SDS-PAGE gel electrophoresis.

**Supplementary Figure S6.** ProE is not a diguanylate cyclase.

**Supplementary Figure S7.** Bacterial two-hybrid analysis of ProE- ProE interaction in vivo.

**Supplementary Figure S8.** ProE can specificly degrade c-di-GMP.

**Supplementary Figure S9.** Analysis the role of residue P315 by homolog model.

**Supplementary Figure S10.** Analysis the role of residues L330 and G527 by homolog model.

| **Table S1**. Bacterial strains and plasmids used in this study | | |
| --- | --- | --- |
| Strain or plasmid | Relevant characteristics | Reference or source |
| ***P. aeruginosa* strains** |  |  |
| PAO1 | Prototrophic laboratory strain | Lab collection |
| ∆*proE* | *proE* in-frame deletion mutant | This study |
| ∆*5294* | *PA5294* in-frame deletion mutant | This study |
| △*rbdA* | *rbdA* in-frame deletion mutant | This study |
| △*bifA* | *bifA* in-frame deletion mutant | This study |
| △*dipA* | *dipA* in-frame deletion mutant | This study |
| ∆*fleQ* | *fleQ* in-frame deletion mutant | This study |
| ∆*pelA* | *pelA* in-frame deletion mutant | This study |
| ∆*pslA* | *pslA* in-frame deletion mutant | This study |
| ∆*proE*∆*pelA* | *proE* and *pelA* double deletion mutant | This study |
| ∆*proE*∆*pslA* | *proE* and *pslA* double deletion mutant | This study |
| ∆*proE*∆*pelA*∆*pslA* | *proE*, *pelA* and *pslA* triple deletion mutant | This study |
| ∆*fleQ*∆*pelA* | *fleQ* and *pelA* double deletion mutant | This study |
| ∆*fleQ*∆*pslA* | *fleQ* and *pslA* double deletion mutant | This study |
| ∆*proE*∆*fleQ* | ∆proE and fleQ double deletion mutant | This study |
| ∆*fleQ*∆*pelA*∆*pslA* | *fleQ*, *pelA* and *pslA* triple deletion mutant | This study |
| PAO1(vc) | PAO1 harboring the empty pBBR1-MCS5 | This study |
| △*proE*(vc) | △*proE* harboring the empty pBBR1-MCS5 | This study |
| △*fleQ*(vc) | △*fleQ* harboring the empty pBBR1-MCS5 | This study |
| PAO1(*proE*) | PAO1 harboring the pBBR1-MCS5-*proE* | This study |
| PAO1(*fleQ*) | PAO1 harboring the pBBR1-MCS5-*fleQ* | This study |
| △*proE* (*proE*) | △*proE* harboring the pBBR1-MCS5-*proE* | This study |
| △*proE* (*fleQ*) | △*proE* harboring the pBBR1-MCS5-*fleQ* | This study |
| △*proE* (*bifA*) | △*proE* harboring the pBBR1-MCS5-*bifA* | This study |
| △*proE*(*rbdA*) | △*proE* harboring the pBBR1-MCS5-*rbdA* | This study |
| △*proE* (*dipA*) | △*proE* harboring the pBBR1-MCS5-*dipA* | This study |
| △*rbdA*(*proE*) | △*rbdA* harboring the pBBR1-MCS5-*proE* | This study |
| △*rbdA*(*bifA*) | △*rbdA* harboring the pBBR1-MCS5-*bifA* | This study |
| △*rbdA*(*rbdA*) | △*rbdA* harboring the pBBR1-MCS5-*rbdA* | This study |
| △*rbdA*(*dipA*) | △*rbdA* harboring the pBBR1-MCS5-*dipA* | This study |
| △*bifA*(*proE*) | △*bifA* harboring the pBBR1-MCS5-*proE* | This study |
| △*bifA*(*bifA*) | △*bifA* harboring the pBBR1-MCS5-bifA | This study |
| △*bifA*(*rbdA*) | △*bifA* harboring the pBBR1-MCS5-*rbdA* | This study |
| △*bifA*(*dipA*) | △*bifA* harboring the pBBR1-MCS5-*dipA* | This study |
| △*dipA*(*proE*) | △*dipA* harboring the pBBR1-MCS5-*proE* | This study |
| △*dipA*(*bifA*) | △*dipA* harboring the pBBR1-MCS5-*bifA* | This study |
| △*dipA*(*rbdA*) | △*dipA* harboring the pBBR1-MCS5-*rbdA* | This study |
| △*dipA*(*dipA*) | △*dipA* harboring the pBBR1-MCS5-*dipA* | This study |
| △*fleQ*(*fleQ*) | △*fleQ* harboring the pBBR1-MCS5-*fleQ* | This study |
| △*fleQ*(*proE*) | △*fleQ* harboring the pBBR1-MCS5-*proE* | This study |
| △*proE*(*proE*-*Q314A*) | △*proE* harboring the pBBR1-MCS5-*proE*(Q314A) | This study |
| △*proE*(*proE*-*P315A*) | △*proE* harboring the pBBR1-MCS5-*proE*(P315A) | This study |
| △*proE*(*proE*-*L330A*) | △*proE* harboring the pBBR1-MCS5-*proE*(L330A) | This study |
| △*proE*(*proE*-*E328A*) | △*proE* harboring the pBBR1-MCS5-*proE*(E328A) | This study |
| △*proE*(*proE*-*R332A*) | △*proE* harboring the pBBR1-MCS5-*proE*(R328A) | This study |
| △*proE*(*proE*-*P343A*) | △*proE* harboring the pBBR1-MCS5-*proE*(P343A) | This study |
| △*proE*(*proE*-*N387A*) | △*proE* harboring the pBBR1-MCS5-*proE*(N387A) | This study |
| △*proE*(*proE*-*E419A*) | △*proE* harboring the pBBR1-MCS5-*proE*(E419A) | This study |
| △*proE*(*proE*-*E422A*) | △*proE* harboring the pBBR1-MCS5-*proE*(E422A) | This study |
| △*proE*(*proE*-*E449A*) | △*proE* harboring the pBBR1-MCS5-*proE*(E449A) | This study |
| △*proE*(*proE*-*D450A*) | △*proE* harboring the pBBR1-MCS5-*proE*(D450A) | This study |
| △*proE*(*proE*-*K470A*) | △*proE* harboring the pBBR1-MCS5-*proE*(K470A) | This study |
| △*proE*(*proE*-*D472A*) | *△proE* harboring the pBBR1-MCS5-*proE*(D472A) | This study |
| △*proE*(*proE*-*E506A*) | *△proE* harboring the pBBR1-MCS5-*proE*(E506A) | This study |
| △*proE*(*proE*-*E509A*) | *△proE* harboring the pBBR1-MCS5-*proE*(E509A) | This study |
| △*proE*(*proE*-*Q526A*) | *△proE* harboring the pBBR1-MCS5-*proE*(E526A) | This study |
| △*proE*(*proE*-*G527A*) | *△proE* harboring the pBBR1-MCS5-*proE*(G527A) | This study |
| PAO1(ProE-GFP) | PAO1 harboring the pBBR1-MCS5-*proE*-*gfp* fusion protein | This study |
| PAO1(RbdA-GFP) | PAO1 harboring the pBBR1-MCS5-*rbdA*-*gfp* fusion protein | This study |
| PAO1(BifA-GFP) | PAO1 harboring the pBBR1-MCS5-*bifA*-*gfp* fusion protein | This study |
| PAO1(DipA-GFP) | PAO1 harboring the pBBR1-MCS5-*dipA*-*gfp* fusion protein | This study |
| PAO1(FleQ-GFP) | PAO1 harboring the pBBR1-MCS5-*fleQ*-*gfp* fusion protein | This study |
| PAO1(pBB-GFP) | PAO1 harboring the pBBR1-MCS5-*gfp* | This study |
| ***E. coli* strains** |  |  |
| DH5α | F-φ80d lacZΔM15 Δ(lacZYA-argF)U169 endA1 recA1 hsdR17(rk-,mk+) supE44λ- thi -1 gyrA96 relA1 phoA | Lab collection |
| BL21(DE3) | F- ompT hsdS(rB-mB-) gal dcm(DE3) | Stratagene |
| XL1-Blue MRF' | Reporter Strain, *Δ(mcrA)183 Δ(mcrCB-hsdSMR-mrr)173*  *endA1 hisB supE44 thi-1 recA1 gyrA96relA1 lac* [F´ lacIq HIS3  aadA Kan^r^ ] | Stratagene |
| XL1-Blue MRF'/pBT-proE  /pTRG-proE | XL1-Blue MRF' harboring plasmids pBT-proE and pTRG-proE | This study |
| XL1-Blue MRF'/pBT  /pTRG-proE | XL1-Blue MRF' harboring plasmids pBT and pTRG-proE | This study |
| XL1-Blue MRF'/pBT-proE  /pTRG | XL1-Blue MRF' harboring plasmids pBT-proE and pTRG | This study |
| XL1-Blue MRF'/pBT-  4608/pTRG-3348 | XL1-Blue MRF' harboring plasmids pBT-4608 and pTRG-3348 | Lab collection |
| **Plasmid** |  |  |
| pK18mobsacB | Broad-host-range gene replacement vector, sacB, Gm^r^ | Lab collection |
| pK18-proE | pK18 containing fragments flanking *proE* fragment; Gm^r^ | This study |
| pK18-5294 | pK18 containing fragments flanking GGDEF coding sequence fragment; Gm^r^ | This study |
| pK18-fleQ | The *fleQ* in-frame deletion cassette in pK18Gm, Gm^R^ | This study |
| pK18-pelA | The *pelA* in-frame deletion cassette in pK18Gm, Gm^R^ | This study |
| pK18-pslA | The *pslA* in-frame deletion cassette in pK18Gm, Gm^R^ | This study |
| pK18-rbdA | The *rbdA* in-frame deletion cassette in pK18Gm, Gm^R^ | This study |
| pK18-bifA | The *bifA* in-frame deletion cassette in pK18Gm, Gm^R^ | This study |
| pK18-dipA | The *dipA* in-frame deletion cassette in pK18Gm, Gm^R^ | This study |
| pBBR1-MCS5 | Broad-host-range expression vector, Gm^r^ | (Kovach et al., 1995) |
| pBBR1-MCS5-proE | *proE* cloned in pBBR1MCS under its native promoter, Gm^r^ | This study |
| pBBR1-MCS5-fleQ | *fleQ* cloned in pBBR1MCS under its native promoter, Gm^r^ | This study |
| pBBR1-MCS5-rbdA | *rbdA* cloned in pBBR1MCS under its native promoter, Gm^r^ | This study |
| pBBR1-MCS5-bifA | *bifA* cloned in pBBR1MCS under its native promoter, Gm^r^ | This study |
| pBBR1-MCS5-dipA | *dipA* cloned in pBBR1MCS under its native promoter, Gm^r^ | This study |
| pBBR1-MCS5-gfp | pBBR1-MCS-5 carrying the fragment of GFP, Gm^r^ | This study |
| pBBR1-MCS5-proE-gfp | pBBR1-MCS-5 carrying the *proE-gfp* fusion protein, Gm^r^ | This study |
| pBBR1-MCS5-fleQ-gfp | pBBR1-MCS-5 carrying the *fleQ-gfp* fusion protein, Gm^r^ | This study |
| pBBR1-MCS5-rbdA-gfp | pBBR1-MCS-5 carrying the *rbdA-gfp* fusion protein, Gm^r^ | This study |
| pBBR1-MCS5-bifA-gfp | pBBR1-MCS-5 carrying the *bifA-gfp* fusion protein, Gm^r^ | This study |
| pBBR1-MCS5-dipA-gfp | pBBR1-MCS-5 carrying the *dipA-gfp* fusion protein, Gm^r^ | This study |
| pBBR1-MCS5-proE (Q314A) | *proE* (Q314A) cloned in pBBR1MCS under its native promoter, Gm^r^ | This study |
| pBBR1-MCS5-proE (P315A) | *proE* (P315A) cloned in pBBR1MCS under its native promoter, Gm^r^ | This study |
| pBBR1-MCS5-proE (L330A) | *proE* (L330A) cloned in pBBR1MCS under its native promoter, Gm^r^ | This study |
| pBBR1-MCS5-proE (E328A) | *proE* (E328A) cloned in pBBR1MCS under its native promoter, Gm^r^ | This study |
| pBBR1-MCS5-proE (R332A) | *proE* (R332A) cloned in pBBR1MCS under its native promoter, Gm^r^ | This study |
| pBBR1-MCS5-proE (P343A) | *proE* (P343A) cloned in pBBR1MCS under its native promoter, Gm^r^ | This study |
| pBBR1-MCS5-proE (N387A) | *proE* (N387A) cloned in pBBR1MCS under its native promoter, Gm^r^ | This study |
| pBBR1-MCS5-proE (E419A) | *proE* (E419A) cloned in pBBR1MCS under its native promoter, Gm^r^ | This study |
| pBBR1-MCS5-proE (E422A) | *proE* (E422A) cloned in pBBR1MCS under its native promoter, Gm^r^ | This study |
| pBBR1-MCS5-proE (D449A) | *proE* (D449A) cloned in pBBR1MCS under its native promoter, Gm^r^ | This study |
| pBBR1-MCS5-proE (D450A) | *proE* (D450A) cloned in pBBR1MCS under its native promoter, Gm^r^ | This study |
| pBBR1-MCS5-proE (K470A) | *proE* (K470A) cloned in pBBR1MCS under its native promoter, Gm^r^ | This study |
| pBBR1-MCS5-proE (D472A) | *proE* (D472A) cloned in pBBR1MCS under its native promoter, Gm^r^ | This study |
| pBBR1-MCS5-proE (E506A) | *proE* (E506A) cloned in pBBR1MCS under its native promoter, Gm^r^ | This study |
| pBBR1-MCS5-proE (E509A) | *proE* (E509A) cloned in pBBR1MCS under its native promoter, Gm^r^ | This study |
| pBBR1-MCS5-proE (Q526A) | *proE* (Q526A) cloned in pBBR1MCS under its native promoter, Gm^r^ | This study |
| pBBR1-MCS5-proE (G527A) | *proE* (G527A) cloned in pBBR1MCS under its native promoter, Gm^r^ | This study |
| pBT | Two-hybrid system bait plasmid containing the *cat* gene, p15A origin of replication and λ cI ORF, Cl^r^ | Stratagene |
| pBT-proE | pBT carrying the full ORF of *proE* gene | This study |
| pTRG | Two-hybrid system target plasmid containing the tet gene,  ColE1 origin of replication, and RNA polymerase α subunit  ORF, Tc^r^ | Stratagene |
| pTRG-proE | pTRG carrying the full ORF of *proE* gene | This study |
| pRK2013 | RK2 derivative, *mob*^+^ *tra*^+^ *ori* ColE1; Kan^r^ | (Figurski and Helinski, 1979) |
| pET28b | His-tag protein expression vector, Kan^r^ | Novagen |
| pET-proE | pET28a containing *proE* | This study |
| pET-rocR | pET28a containing *rocR* | This study |
| pET-wspR | pET28a containing *wspR* | This study |
| pET-proE(Q314A) | *proE*(Q314A) cloned in vector pET28b for protein purification | This study |
| pET-proE(P315A) | *proE*(P315A) cloned in vector pET28b for protein purification | This study |
| pET-proE(L330A) | *proE*(L330A) cloned in vector pET28b for protein purification | This study |
| pET-proE(E328A) | *proE*(P328A) cloned in vector pET28b for protein purification | This study |
| pET-proE(R332A) | *proE*(R332A) cloned in vector pET28b for protein purification | This study |
| pET-proE(P343A) | *proE*(P343A) cloned in vector pET28b for protein purification | This study |
| pET-proE(N387A) | *proE*(N387A) cloned in vector pET28b for protein purification | This study |
| pET-proE(E419A) | *proE*(E419A) cloned in vector pET28b for protein purification | This study |
| pET-proE(E422A) | *proE*(E422A) cloned in vector pET28b for protein purification | This study |
| pET-proE(D449A) | *proE*(D449A) cloned in vector pET28b for protein purification | This study |
| pET-proE(D450A) | *proE*(D450A) cloned in vector pET28b for protein purification | This study |
| pET-proE(K470A) | *proE*(K470A) cloned in vector pET28b for protein purification | This study |
| pET-proE(D472A) | *proE*(D472A) cloned in vector pET28b for protein purification | This study |
| pET-proE(E506A) | *proE*(E506A) cloned in vector pET28b for protein purification | This study |
| pET-proE(E509A) | *proE*(E509A) cloned in vector pET28b for protein purification | This study |
| pET-proE(Q526A) | *proE*(Q526A) cloned in vector pET28b for protein purification | This study |
| pET-proE(G527A) | *proE*(G527A) cloned in vector pET28b for protein purification | This study |

*Symbol: Gm^r^, gentamicin resistant; Cml^r^, chloramphenicol resistant; Tc^r^, tetracycline resistant; Kan^r^, kanamycin resistant.

**Table S2.** PCR primers used in this study

| Primer | Sequence (5’-3’) |
| --- | --- |
| For in-frame deletion |  |
| proE-Up-F | ctatgacatgattacgaattcTTCGTGTTCCTTGCGGATG |
| proE-Up-R | tcggcgctcaattgccgagTGCGGGCGCTGACAAGCA |
| proE-Down-F | aCTCGGCAATTGAGCGCCG |
| proE-Down-R | caggtcgactctagaggatccTGTGCATGGGCATCATCGG |
| PA5294-Up-F | ctatgacatgattacgaattcTGGCGTCGTCCACACGCA |
| PA5294-Up-R | gaGAAGACCATGGCGACATAGGC |
| PA5294-Down-F | tatgtcgccatggtcttcTCGGCGCTCAATTGCCGA |
| PA5294-Down-R | caggtcgactctagaggatccTCCGCCAGTTCCAGGACAG |
| fleQ-Up-F | ctatgacatgattacgaattcGGTGAGCTGGATCAGGTCTGTC |
| fleQ -Up-R | cgacctgtcaatcCCACATTTTGATCAGCTGCCT |
| fleQ-Down-F | aatgtggGATTGACAGGTCGTTTCGCAA |
| fleQ-Down-R | caggtcgactctagaggatccGGGCGATCACCTCACGCC |
| pelA-Up-F | ctatgacatgattacgaattcTCGTTGGCCGGAAAAGACA |
| pelA-Up-R | ttgtcagcggcaCCGCATGCCCAGCCTACG |
| pelA-Down-F | gcatgcggTGCCGCTGACAAGCATCC |
| pelA-Down-R | caggtcgactctagaggatccCCGGCCAGTTCGGCAAAG |
| pslA-Up-F | ctatgacatgattacgaattcCGAACGGCCAACCTGCGT |
| pslA-Up-R | gcgttcatcagtagacATGCATGTTGTTTGCTCTGCC |
| pslA-Down-F | gcatGTCTACTGATGAACGCCGTCG |
| pslA-Down-R | caggtcgactctagaggatccCTCCGGGGTGATGCCGAA |
| rbdA-Up-F | ctatgacatgattacgaattcCCCTGCACCAGGCGCTCG |
| rbdA -Up-R | agcgcttccatgacttGCACCGCGGAGAGGACGT |
| rbdA -Down-F | gtgcAAGTCATGGAAGCGCTGCG |
| rbdA -Down-R | caggtcgactctagaggatccATGGCCACCGCCAGTGAG |
| bifA-Up-F | ctatgacatgattacgaattcGGAACCACACCTTCTACTGGAACT |
| bifA -Up-R | agcTAGGCGTCGAAGACGATCTGC |
| bifA -Down-F | atcgtcttcgacgcctaGCTGCCGGCCAGGGAACT |
| bifA -Down-R | caggtcgactctagaggatccCGAGGTGCGACCCTTGACC |
| dipA-Up-F | ctatgacatgattacgaattcTACCTGCGCCGCTCCCCA |
| dipA -Up-R | tgtccttgaAGAGGGTAGTCGCCGGCA |
| dipA -Down-F | gactaccctctTCAAGGACATCCCGGACAGC |
| dipA -Down-R | caggtcgactctagaggatccTGTAGCCGCCGGCATAAC |
| For *in trans* complementation |  |
| proE-F | gataagcttgatatcgaattcGGTCAGGCCCTTGCCCTC |
| proE -R | cgctctagaactagtggatccTCAATTGCCGAGCGGCTG |
| fleQ-F | gataagcttgatatcgaattcATGTGGCGCGAAACCAAA |
| fleQ -R | cgctctagaactagtggatccTCAATCATCCGACAGGTCGTC |
| rbdA-F | gataagcttgatatcgaattcATGAGGCAGAACCGGACTCTC |
| rbdA-R | cgctctagaactagtggatccCTACCGGAGGTTCTGTCCCAG |
| bifA-F | gataagcttgatatcgaattcCACCAAGACTTCCGTCGGC |
| bifA-R | cgctctagaactagtggatccTCAGGGCCGTTCGCTGCT |
| dipA-F | gataagcttgatatcgaattcATGAAAAGTCATCCCGATGCC |
| dipA -R | cgctctagaactagtggatccTCAGTGCAGGGTGCGGCA |
| For protein expression |  |
| pET-proE-F | acagcaaatgggtcgggatccGTTGTCAGCGCCCGCAGA |
| pET-proE-R | ctcgagtgcggccgcaagcttTCAATTGCCGAGCGGCTG |
| pET-rocR-F | taagaaggagatataccatggGCATGAATGATTTGAATGTTCTGG |
| pET- rocR -R | tggtggtgctcgagtgcggccgcGGATCCGGAGCAATAGTCGAG |
| pET-wspR-F | gtgccgcgcggcagccatatgATGCACAACCCTCATGAGAGCA |
| pET-wspR-R | ctcgagtgcggccgcaagcttGCCCGCCGGGGCCGGCGG |
| For bacterial two-hybrid |  |
| pBT-proE-F | gaagagacgtttggcgcggccgcATTGTCAGCGCCCGCAGA |
| pBT-proE-R | aattaattaactcgaggatccTCAATTGCCGAGCGGCTG |
| pTRG-proE-F | aaaccagaggcggccggatccTTGTCAGCGCCCGCAGAA |
| pTRG-proE-R | gcgccagctcagactgaattcTCAATTGCCGAGCGGCTG |
| For expression GFP fusion protein |  |
| ProE-GFP-Up-F | gataagcttgatatcgaattcGGTCAGGCCCTTGCCCTC |
| ProE-GFP-Up-R | tcctcgcccttgctcacATTGCCGAGCGGCTGGCC |
| ProE-GFP-Down-F | aatGTGAGCAAGGGCGAGGAGC |
| ProE-GFP-Down-R | cttctagaactagttggatccTTACTTGTACAGCTCGTCCATGCC |
| FleQ-GFP-Up-F | gataagcttgatatcgaattcAGGCCTTGCGCGTGAGCC |
| FleQ-GFP-Up-R | gataagcttgatatcgaattcAGGCCTTGCGCGTGAGCC |
| FleQ-GFP-Down-F | gataagcttgatatcgaattcAGGCCTTGCGCGTGAGCC |
| FleQ-GFP-Down-R | gataagcttgatatcgaattcAGGCCTTGCGCGTGAGCC |
| RbdA-GFP-Up-F | gataagcttgatatcgaattcCGGCGACCACCAGCAACT |
| RbdA-GFP-Up-R | cttgctcacCCGGAGGTTCTGTCCCAGG |
| RbdA-GFP-Down-F | agaacctccggGTGAGCAAGGGCGAGGAGC |
| RbdA-GFP-Down-R | cttctagaactagttggatccTTACTTGTACAGCTCGTCCATGCC |
| BifA-GFP-Up-F | gataagcttgatatcgaattcCTCCCTGGCCCTGGCCAG |
| BifA-GFP-Up-R | ttgctcacGGGCCGTTCGCTGCTGGT |
| BifA-GFP-Down-F | agcgaacggcccGTGAGCAAGGGCGAGGAGC |
| BifA-GFP-Down-R | cttctagaactagttggatccTTACTTGTACAGCTCGTCCATGCC |
| DipA-GFP-Up-F | gataagcttgatatcgaattcGCGAGCATGCCACGCTCG |
| DipA -GFP-Up-R | ccttgctcacGTGCAGGGTGCGGCAGGG |
| DipA -GFP-Down-F | caccctgcacGTGAGCAAGGGCGAGGAGC |
| DipA -GFP-Down-R | cgctctagaactagtggatccTTACTTGTACAGCTCGTCCATGCC |
| For Point Mutation |  |
| proE-Q314A-F | TGGAGCTGCACTACgccCCGCGACTGTGCCTGGAC |
| proE-Q314A-R | ggcGTAGTGCAGCTCCAGCTCGTCGCGACGCA |
| proE-P315A-F | CTACCAGgccCGACTGTGCCTGGACAGCGGGC |
| proE- P315A -R | ACAGTCGggcCTGGTAGTGCAGCTCCAGCTCG |
| proE-L330A-F | GGAGGCGgccGTGCGCTGGCGCCACGGCGAGC |
| proE-L330A -R | AGCGCACggcCGCCTCCAGGCCGACGATGCGC |
| proE-E328A-F | ATCGTCGGCCTGgccGCGCTGGTGCGCTGGCGC |
| proE-E328A -R | GCggcCAGGCCGACGATGCGCCCGCTGTCCAG |
| proE-R332A-F | CTGGTGgccTGGCGCCACGGCGAGCGGGGCCT |
| proE-R332A -R | TGGCGCCAggcCACCAGCGCCTCCAGGCCGAC |
| proE-P343A-F | CTCACGgccAGCGAGTTCGTCCCGCTGGCCGA |
| proE-P343A -R | AACTCGCTggcCGTGAGCAGGCCCCGCTCGCC |
| proE-N387A-F | GCGGTGgccCTGTCGTTCCGCCAGTTCCAGGA |
| proE-N387A-R | AACGACAGggcCACCGCCATGTGCAACGGCTC |
| proE-E419A-F | TCGAGTTCgccCTGACCGAGACCGCCGTGATG |
| proE-E419A-R | GGTCAGggcGAACTCGAGCCAGCGCGCGTCGA |
| proE-D449A-F | TTTTCGCTGgccGACTTCGGCACCGGTTTCTC |
| proE-D449A-R | AAGTCggcCAGCGAAAAGCGCACGCCCAGTTG |
| proE-D450A-F | TTTTCGCTGGACgccTTCGGCACCGGTTTCTCG |
| proE-D450A-R | AAggcGTCCAGCGAAAAGCGCACGCCCAGTTG |
| proE-K470A-F | ATCACCCTGTTGgccATCGACCGCAGCTTCGTCG |
| proE-K470A -R | ATggcCAACAGGGTGATCGGCAGGCTATTGAG |
| proE-D472A-F | AAGATCgccCGCAGCTTCGTCGGCGGCATGGA |
| proE-D472A-R | AAGCTGCGggcGATCTTCAACAGGGTGATCGGC |
| proE-E506A-F | TGGAAGTGGTTGCCgccGGCGTGGAGACCCTGCGC |
| proE-E506A-R | ggcGGCAACCACTTCCAGGTTGAGGTTGTGCG |
| proE-E509A-F | AAGGCGTGgccACCCTGCGCCAGCAGGAGCAG |
| proE-E509A-R | CAGGGTggcCACGCCTTCGGCAACCACTTCCA |
| proE-Q526A-F | GTGgccGGCTACTGGATCAGTCCGCCGCTGCC |
| proE-Q526A-R | ATCCAGTAGCCggcCACCTGGTCGCAACCGAA |
| proE-G527A-F | AGgccTACTGGATCAGTCCGCCGCTGCCGTTG |
| proE- G527A -R | ACTGATCCAGTAggcCTGCACCTGGTCGCAACC |
| For qRT-PCR |  |
| pelA-F | CCTTCAGCCATCCGTTCTTCT |
| pelA-R | TCGCGTACGAAGTCGACCTT |
| pslA-F | AAGATCAAGAAACGCGTGGAAT |
| pslA-R | TGTAGAGGTCGAACCACACCG |
| rplU-F | GCAGCACAAAGTCACCGAAG |
| rplU-R | CCGATTTTCACGTCTTCGCC |

**Table S3.** Analysis of the enzyme activity of ProE and its variant.

| Protein | Enzyme activity | Residue role (interaction) ^a^ | Reference |
| --- | --- | --- | --- |
| WT | 100% |  |  |
| Q314 | 44.8% |  | (Rao et al., 2008) |
| P315 | 0 |  |  |
| E328 | 3.6% | Me1 | (Tamayo et al., 2005;Rao et al., 2008;Tchigvintsev et al., 2010;Yang et al., 2017) |
| L330 | 8.5% |  |  |
| R332 | 4.9% | P2 | (Rao et al., 2008;Tchigvintsev et al., 2010;Yang et al., 2017) |
| P343 | 29.6% |  |  |
| N387 | 0 | Me1, P1 | (Rao et al., 2008;Tchigvintsev et al., 2010;Yang et al., 2017) |
| E419 | 0 | Me1 | (Rao et al., 2008;Tchigvintsev et al., 2010;Yang et al., 2017) |
| E422 | 0 |  | (Rao et al., 2008) |
| D449 | 5.1% | Me1, Me2, dimerization | (Rao et al., 2008;Tchigvintsev et al., 2010;Yang et al., 2017) |
| D450 | 0 | Me2, dimerization | (Rao et al., 2008;Tchigvintsev et al., 2010;Yang et al., 2017) |
| K470 | 0 | Water-1, E419 | (Rao et al., 2008;Tchigvintsev et al., 2010;Yang et al., 2017) |
| D472 | 37% |  | (Rao et al., 2008) |
| E506 | 0 | Me2 | (Rao et al., 2008;Tchigvintsev et al., 2010;Yang et al., 2017) |
| E509 | 53.7% |  | (Rao et al., 2008) |
| Q526 | 6.7% | E328, K470 | (Rao et al., 2008;Tchigvintsev et al., 2010;Yang et al., 2017) |
| G527 | 1.4% |  |  |

^a^Me1: metal ion-1; P2: c-di-GMP phosphate-2; G1: c-di-GMP guanine base-1; P1: c-di- GMP phosphate-1; Water-1: catalytic water molecule-1.

**Supplementary Figure S1**

**
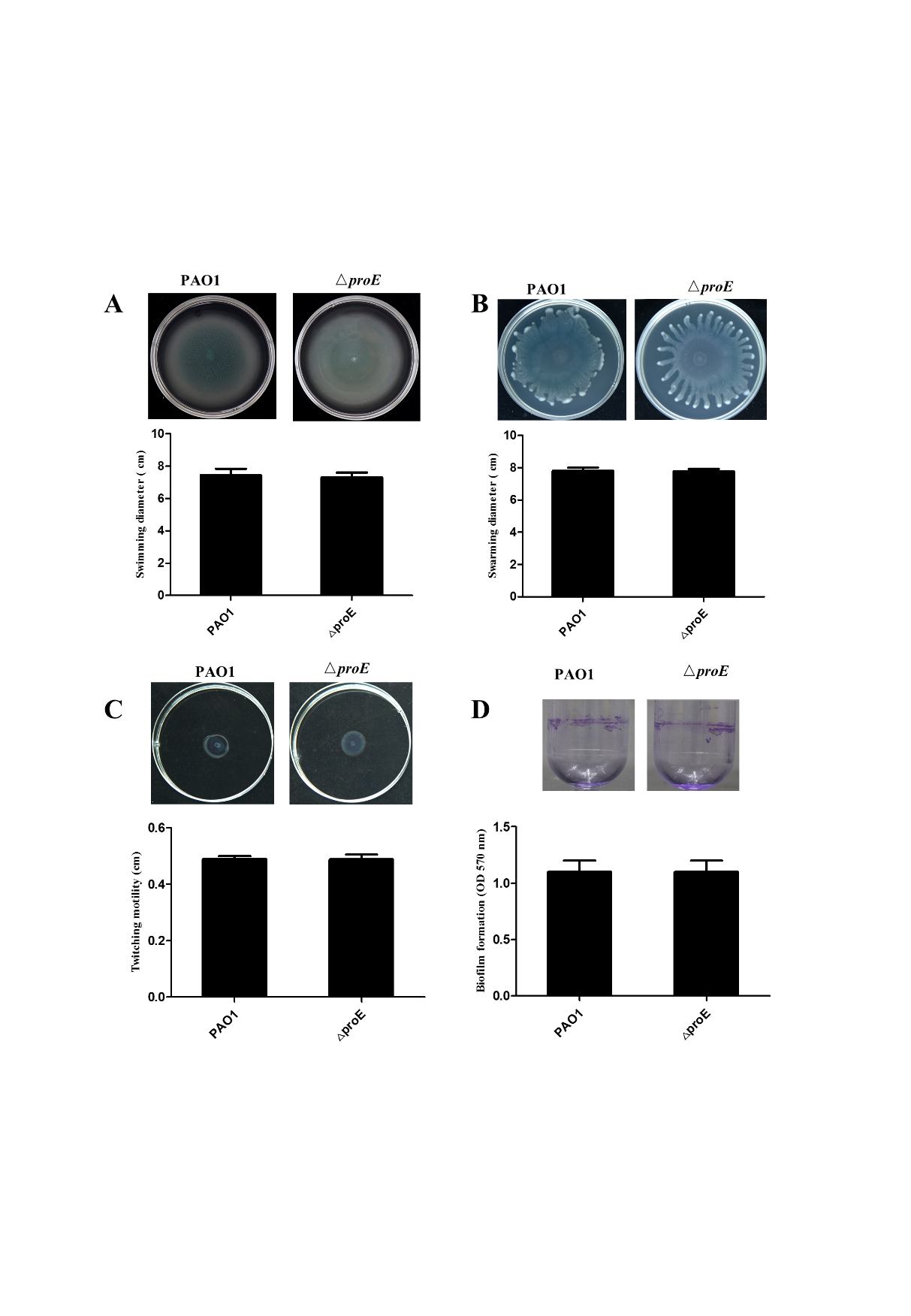
**

**Supplementary Figure S1. ProE doesn’t regulate motility and biofilm formation.** (A) Swimming motility; (B) Swarming motility; (C) Twitching motility; (D) Biofilm formation. The data are means of three replicates and error bars indicate standard deviation.

**Supplementary Figure S2**

**
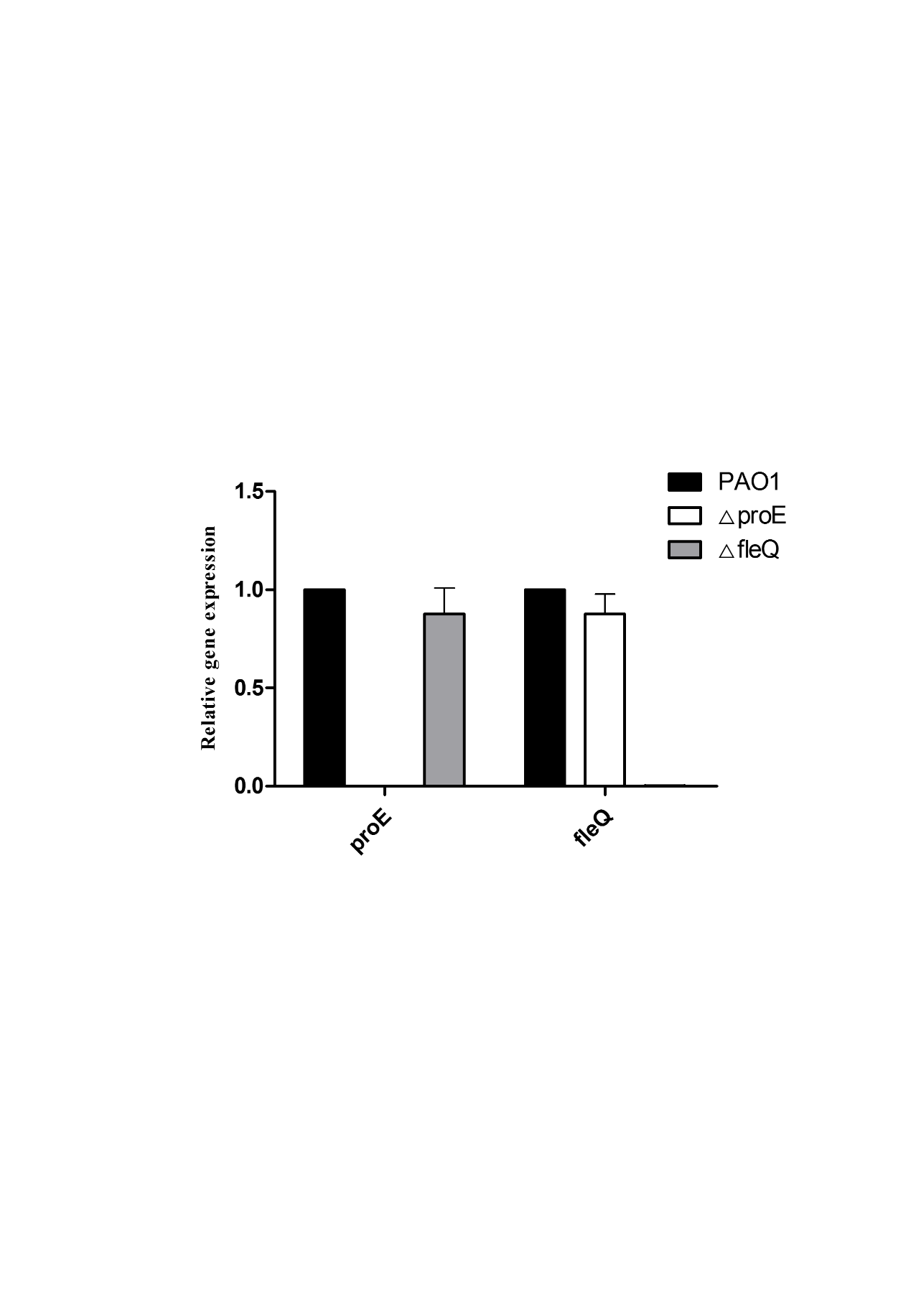
**

**Supplementary Figure S2. *proE* and *fleQ* are not transcriptionally regulated by each other.** The relative gene expression of proE and fleQ in PAO1, △proE, △fleQ, by qRT-PCR analysis. The data are means of three replicates and error bars indicate standard deviation.

**Supplementary Figure S3**

**
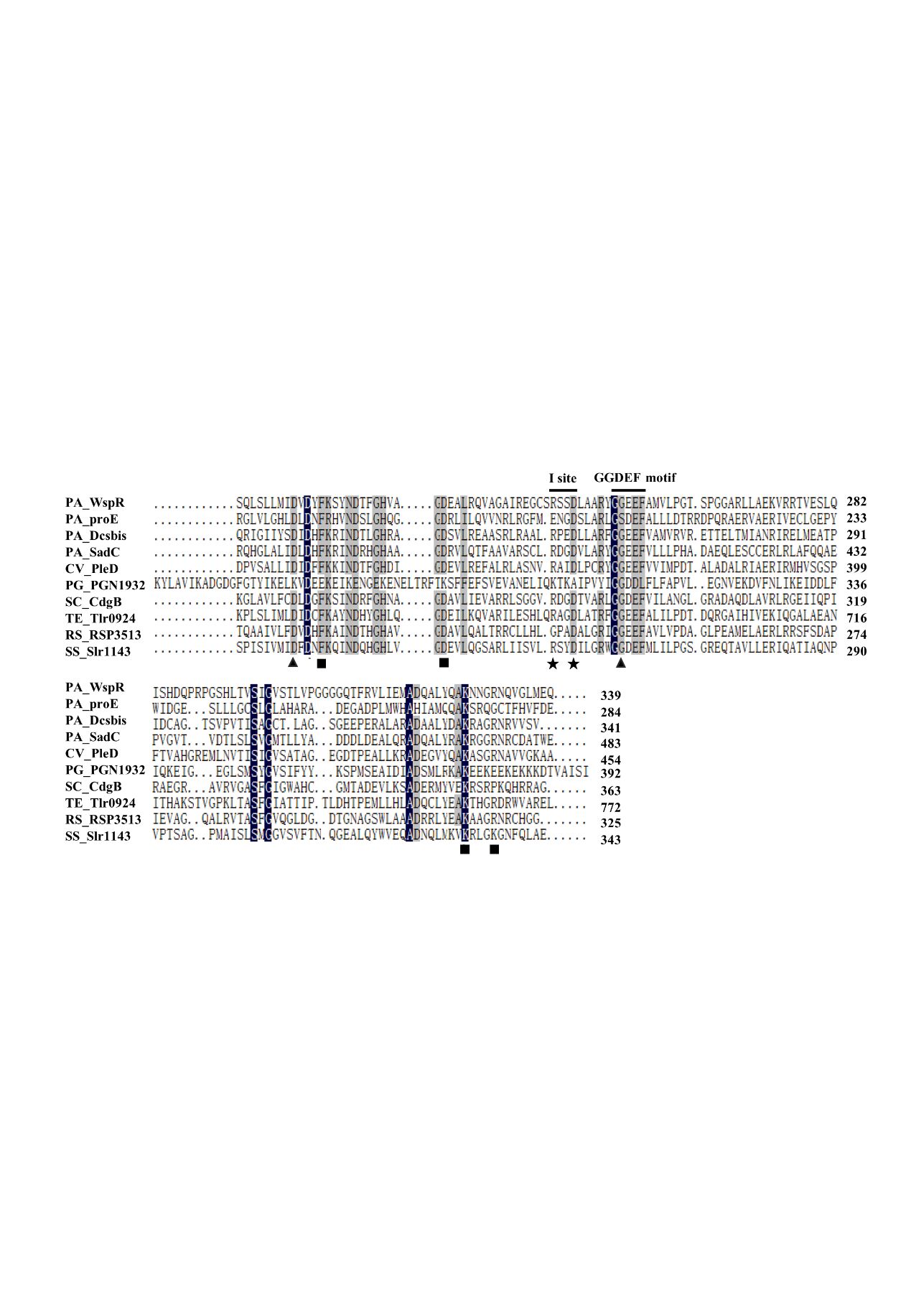
**

**Supplementary Figure S3. Sequence alignment of ProE GGDEF domain with other GGDEF-containing diguanylate cyclase.** The amino acid sequence of WspR (Q9HXT9), ProE (Q9HTQ9), Dcsbis (Q9I072), SadC (Q9HW69) from *P. aeruginosa*，PleD (B8GZM2) from *Caulobacter vibrioides*, PGN1932 (B2RM56) from *Porphyromonas gingivalis*, CdgB (Q9KXW0) from *Streptomyces coelicolor*, Tlr0924 (Q8DKD5) from *Thermosynechococcus elongatus.* RSP3513 (Q3IWG8) from *Rhodobacter sphaeroides,* Slr1143 (P73272) from *Synechocystis* sp. The amino acids highlighted with black stands for 100% similarity, and grey indicates similarity level ≥75%. D^160^ and D^203^ (filled triangle) are involved in Mg^2+^ binding , F^164^, D^177^, K^272^, R^276^ (filled square) are involved in GTP binding, E^192^ and D^195^ (asterisks) were reported to bind to c-di-GMP.

**Supplementary Figure S4**

**
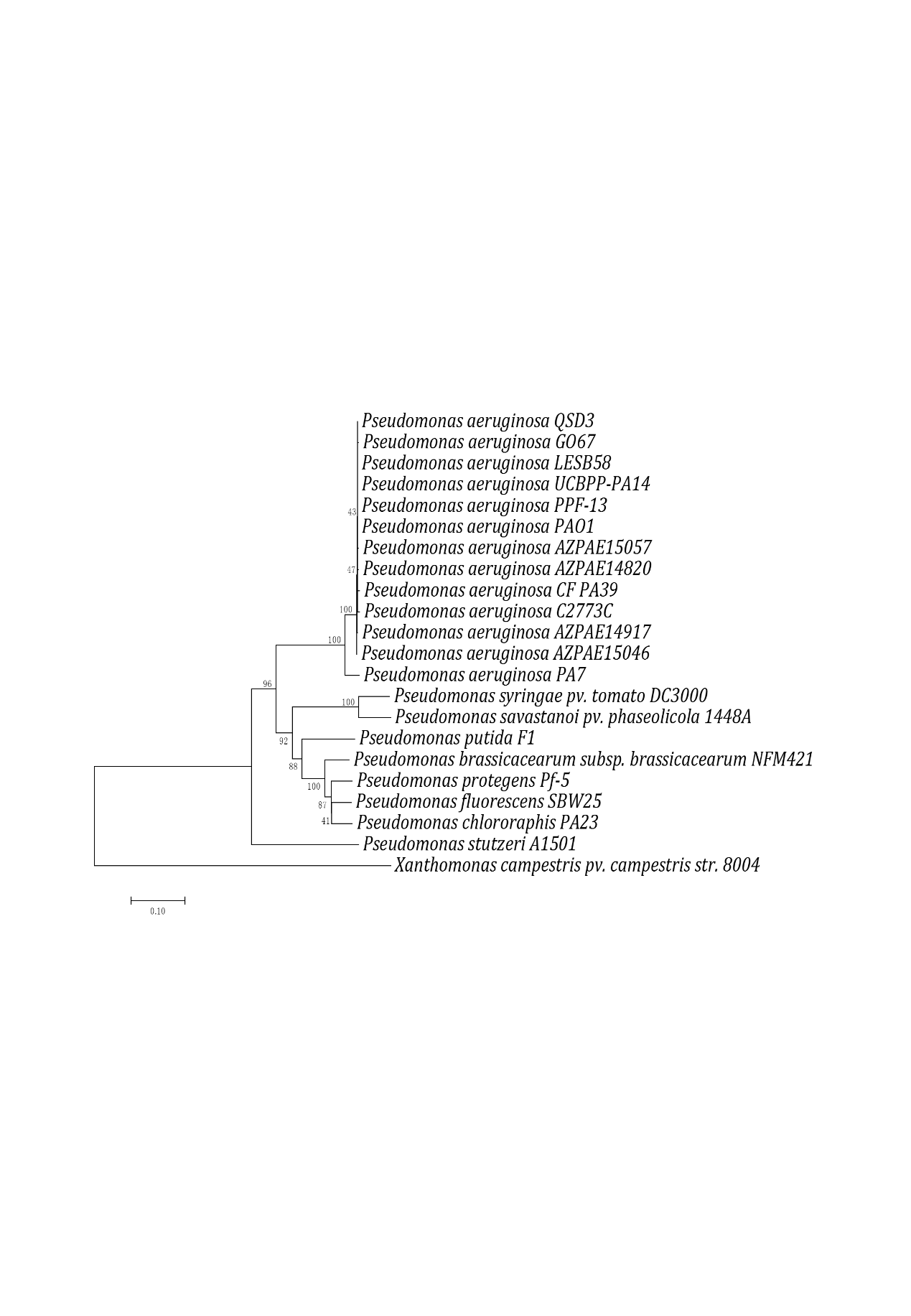
**

**Supplementary Figure S4.** Phylogenetic analysis of ProE proteins from several *Pseudomonas* strains.

**Supplementary Figure S5**


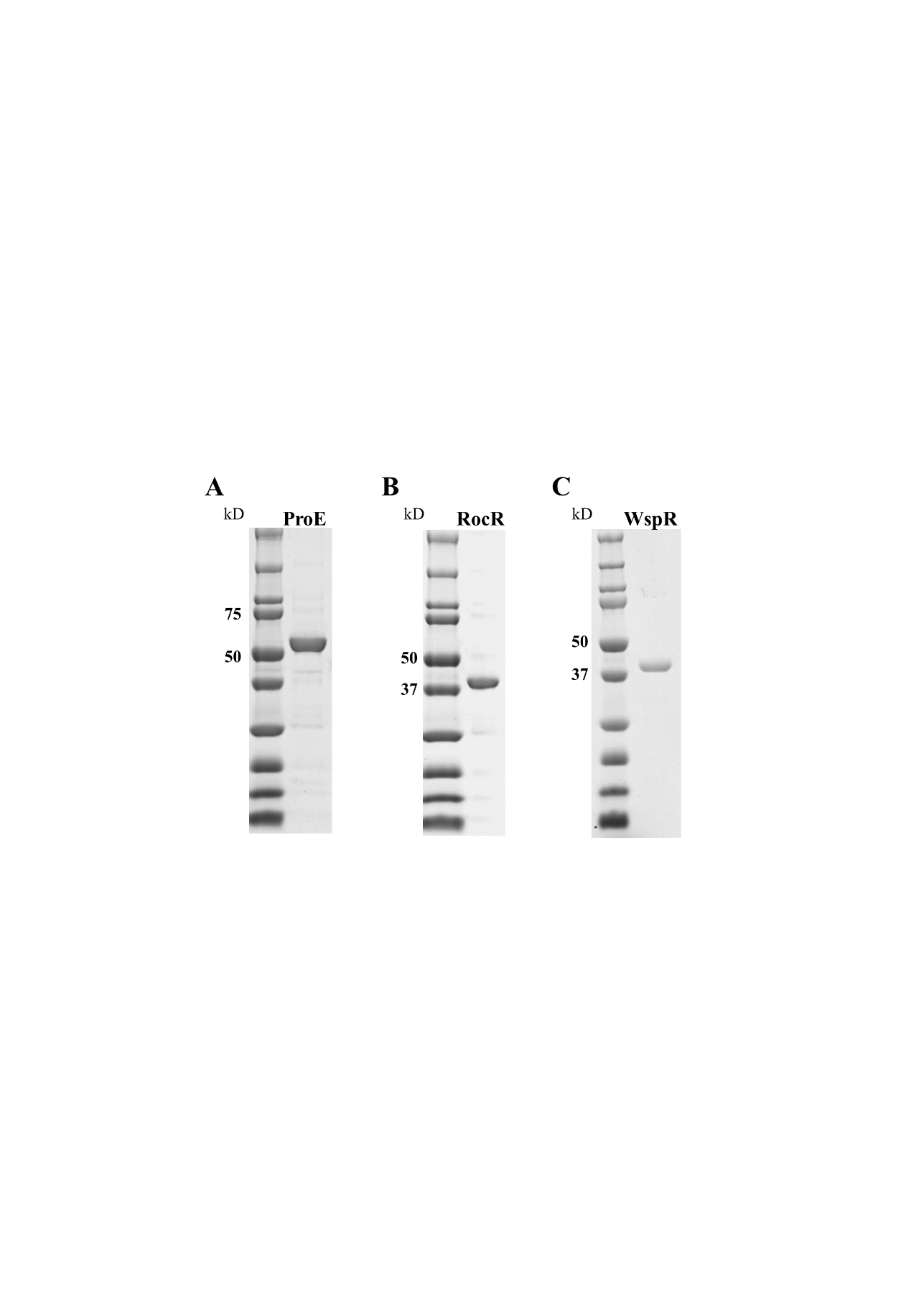


**Supplementary Figure S5.** SDS-PAGE gel electrophoresis. Purified ProE (A), RocR (B) and WspR (C) proteins.

**Supplementary Figure S6**


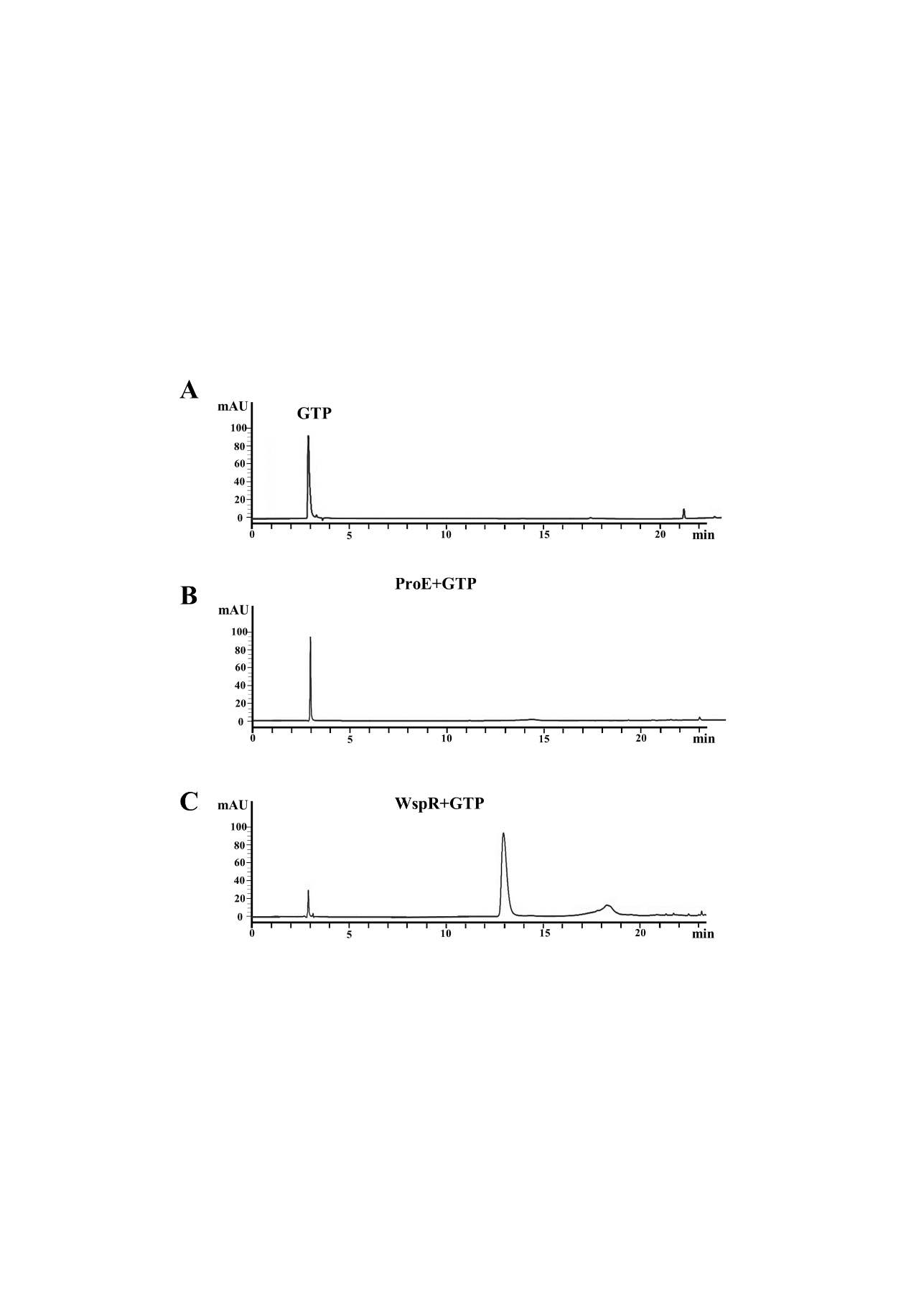


**Supplementary Figure S6. ProE is not a diguanylate cyclase. (**A) Standards of GTP. (B-C) after incubation with ProE (B) or WspR (C) at 37°C for 2 h, the reaction mix was analyzed by HPLC.

**Supplementary Figure S7**

**
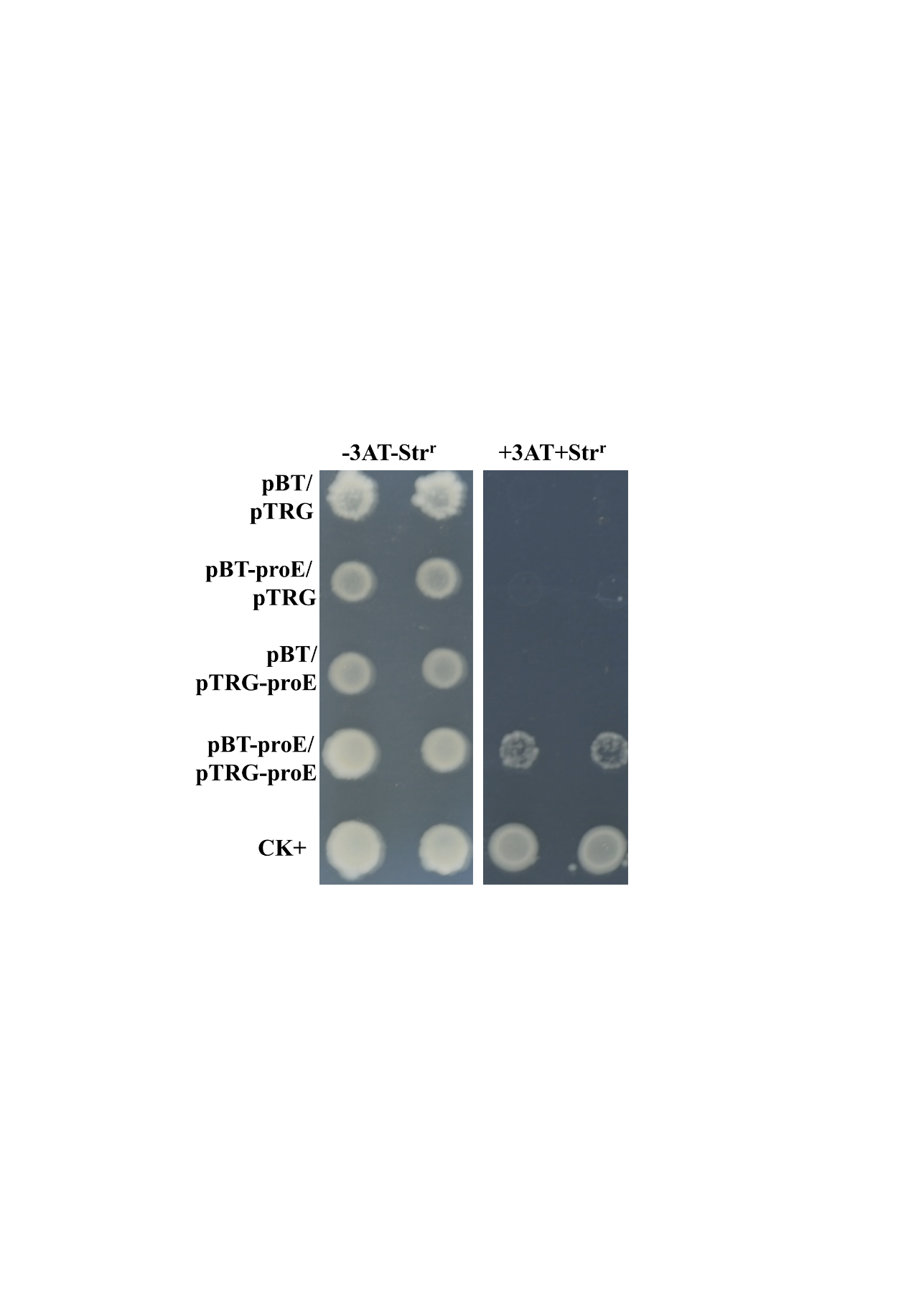
**

**Supplementary Figure S7.** Bacterial two-hybrid analysis of ProE-ProE interaction in vivo.

**Supplementary Figure S8**

**
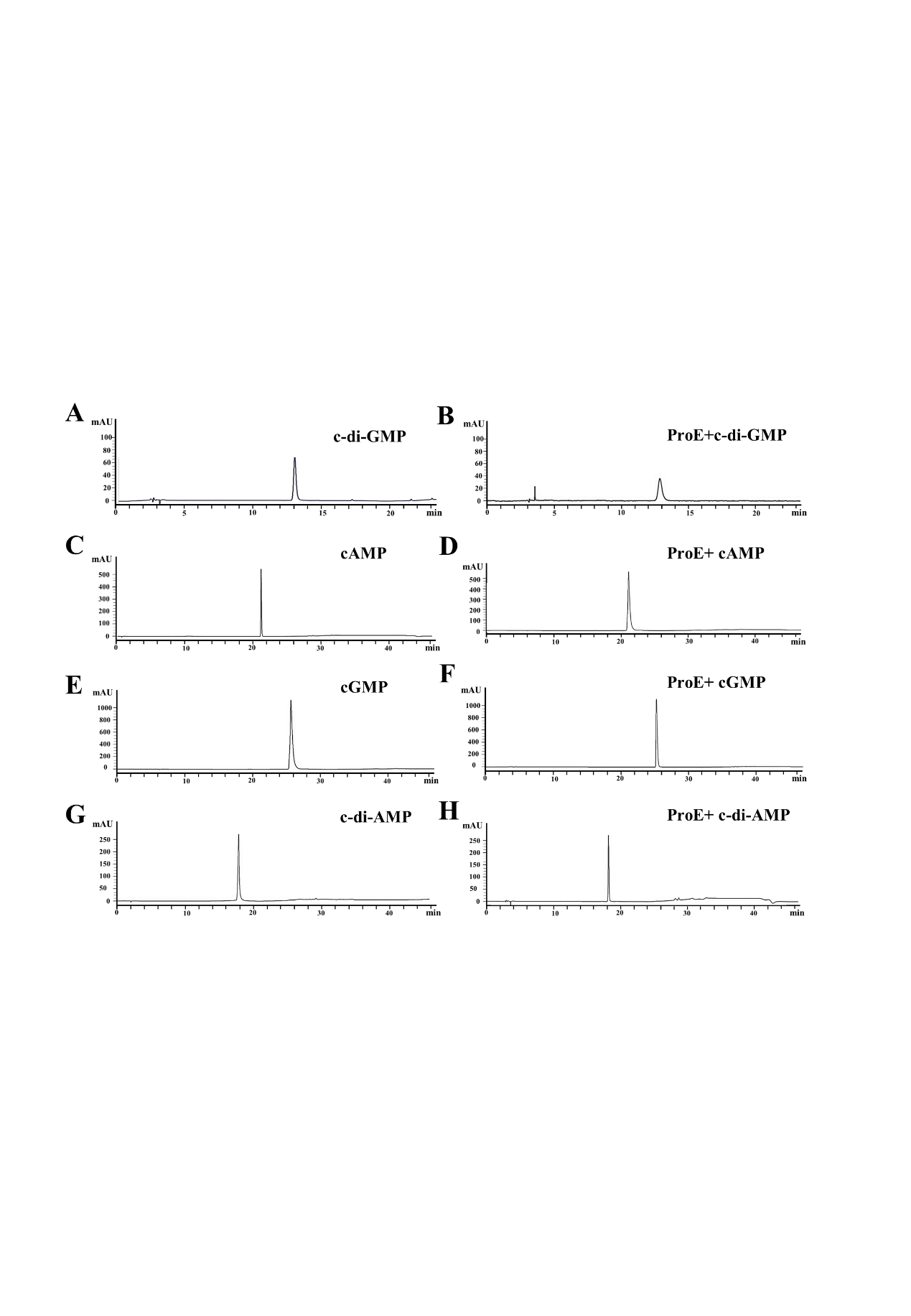
**

**Supplementary Figure S8. ProE can specifically degrade c-di-GMP.** The standards of **(**A)c-di-GMP, (C) cAMP, (E) cGMP, (G) c-di-AMP in reaction buffer, (B), (D), (F), (H) after incubation with ProE at 37°C for 20 min, the reaction mix was analyzed by HPLC.

**Supplementary Figure S9**


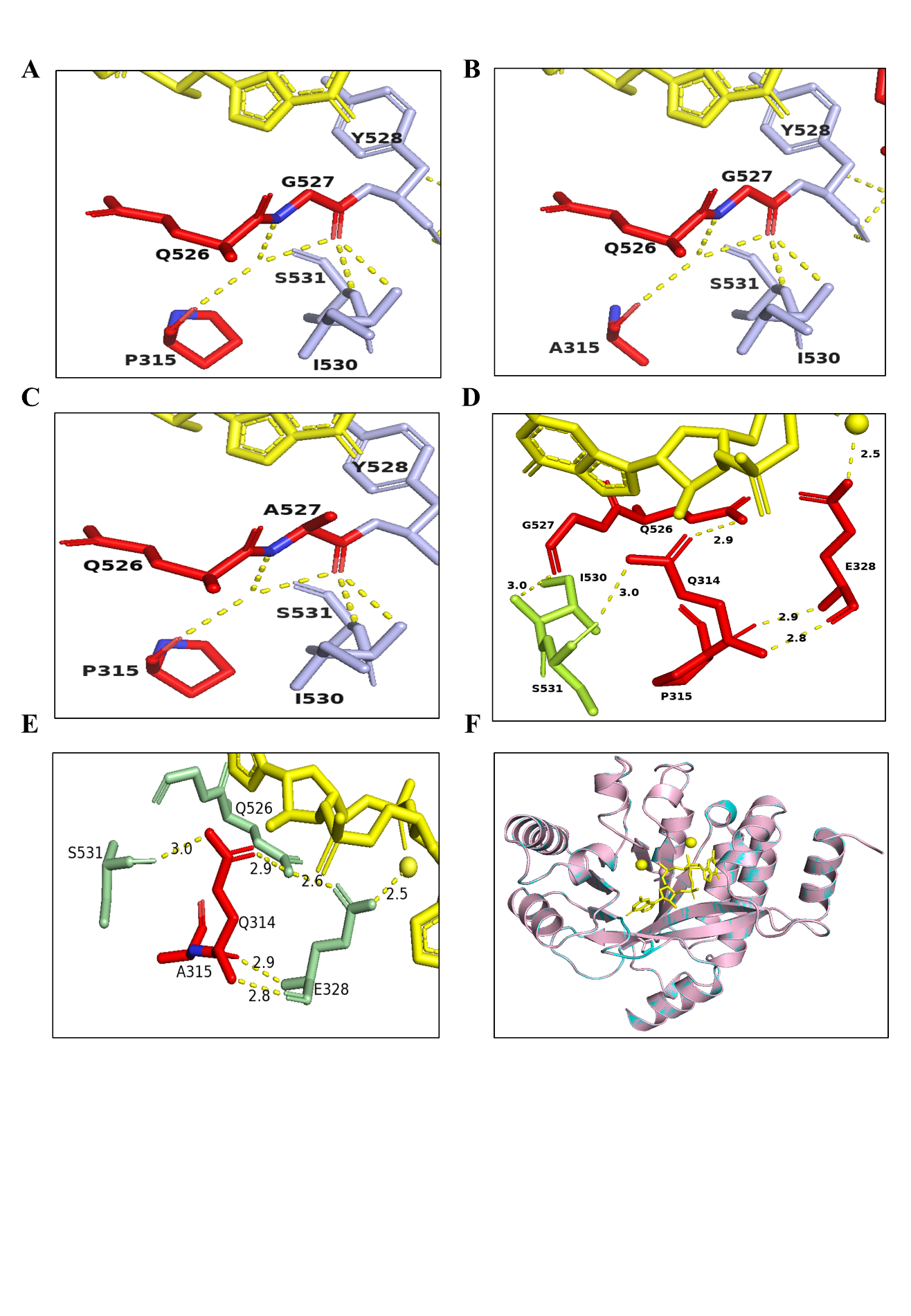


**Supplementary Figure S9. Analysis the role of residue P315 by homolog model.** The water-mediated hydrogen bond interaction between P315 and G527 **(A)**, P315A mutant with G527 **(B)** and P315 and G527A **(C)**. The polar interaction between Q314 **(D)** with E328, Q526 and S531 were not disrupted by P315A mutations **(E)**. **(F)** Shows the superposed ProE models of wild type ProE and mutant P315A ProE. The wild type and mutant ProE models were generated from the 4Y9P (Ca2^+^- c-di-GMP) template and are represented as cyan and pink cartoons respectively. The c-di-GMP and calcium ions are shown as yellow sticks and yellow spheres, respetively.

**Supplementary Figure S10**


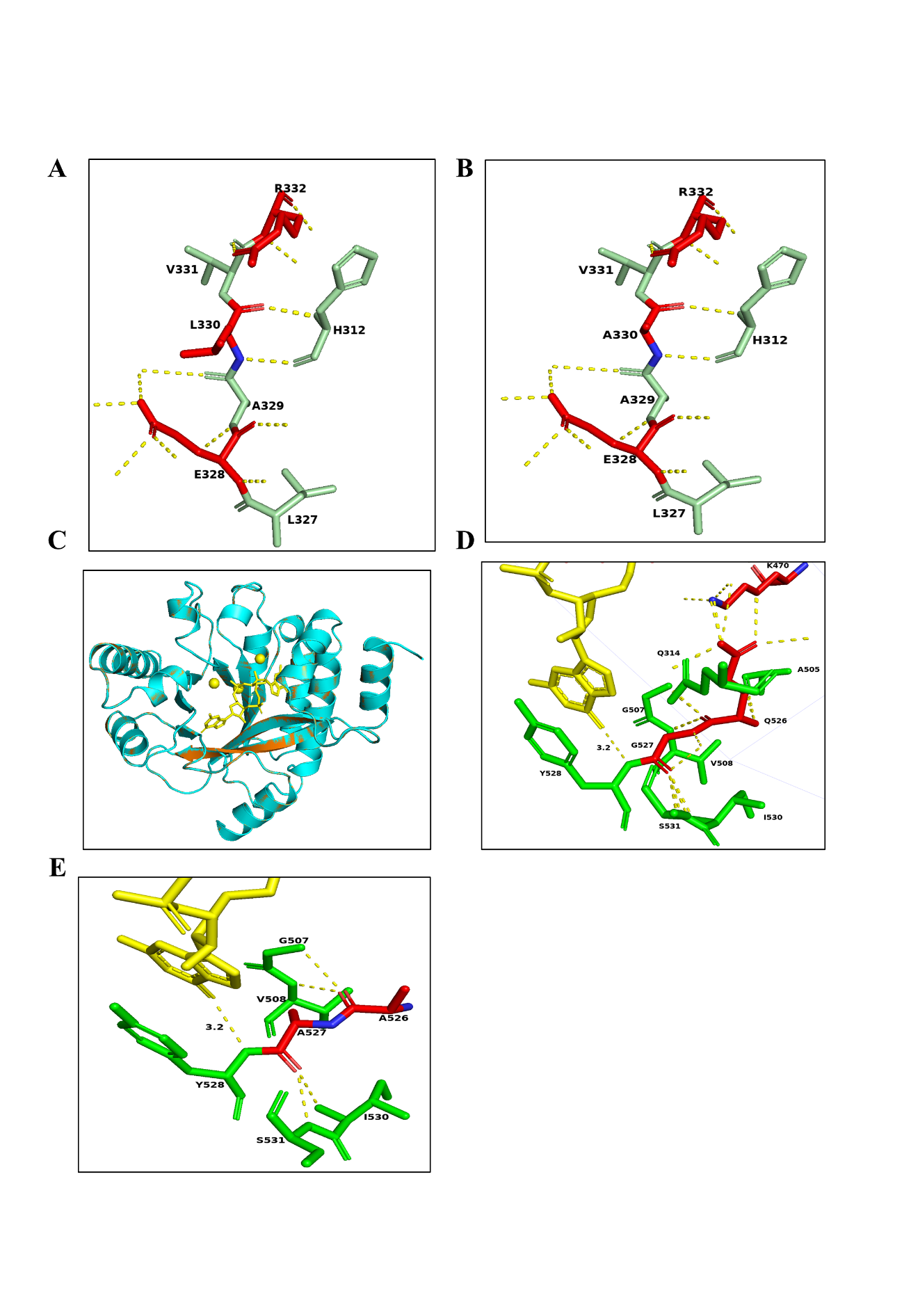


**Supplementary Figure S10. Analysis the role of residues L330 and G527 by homolog model.** Polar interaction between L330 and H312 **(A)** were not abolished despite L330A mutation **(B)**. **(C)** Superposed structure of L330 (cyan) and and L330A (orange) ProE models. The models were generated from the 4Y9P template. The interaction of conserved residues G527 and Q526 and **(D)** G527A and Q526A mutants **(E)**. The c-di-GMP and calcium ions are shown as yellow sticks and spheres, respetively. Polar interaction are shown as yellow dash lines.

**References:**

Figurski, D.H., and Helinski, D.R. (1979). Replication of an origin-containing derivative of plasmid RK2 dependent on a plasmid function provided in trans. *Proc Natl Acad Sci U S A* 76**,** 1648-1652.

Kovach, M.E., Elzer, P.H., Hill, D.S., Robertson, G.T., Farris, M.A., Roop, R.M., 2nd, and Peterson, K.M. (1995). Four new derivatives of the broad-host-range cloning vector pBBR1MCS, carrying different antibiotic-resistance cassettes. *Gene* 166**,** 175-176.

Rao, F., Yang, Y., Qi, Y., and Liang, Z.X. (2008). Catalytic mechanism of cyclic di-GMP-specific phosphodiesterase: a study of the EAL domain-containing RocR from Pseudomonas aeruginosa. *J Bacteriol* 190**,** 3622-3631.

Tamayo, R., Tischler, A.D., and Camilli, A. (2005). The EAL domain protein VieA is a cyclic diguanylate phosphodiesterase. *J Biol Chem* 280**,** 33324-33330.

Tchigvintsev, A., Xu, X., Singer, A., Chang, C., Brown, G., Proudfoot, M., Cui, H., Flick, R., Anderson, W.F., Joachimiak, A., Galperin, M.Y., Savchenko, A., and Yakunin, A.F. (2010). Structural insight into the mechanism of c-di-GMP hydrolysis by EAL domain phosphodiesterases. *J Mol Biol* 402**,** 524-538.

Yang, C., Cui, C., Ye, Q., Kan, J., Fu, S., Song, S., Huang, Y., He, F., Zhang, L.H., Jia, Y., Gao, Y.G., Harwood, C.S., and Deng, Y. (2017). Burkholderia cenocepacia integrates cis-2-dodecenoic acid and cyclic dimeric guanosine monophosphate signals to control virulence. *Proc Natl Acad Sci U S A* 114**,** 13006-13011.
